# Quantification of beta-cell carrying capacity in prediabetes

**DOI:** 10.1371/journal.pcbi.1014589

**Published:** 2026-09-15

**Authors:** Aurore Woller, Yuval Tamir, Alon Bar, Avi Mayo, Michal Rein, Anastasia Godneva, Netta Mendelson Cohen, Eran Segal, Yoel Toledano, Smadar Shilo, Didier Gonze, Uri Alon

**Affiliations:** 1 Department of Molecular Cell Biology, Weizmann Institute of Science, Rehovot, Israel; 2 Unité de Chronobiologie théorique, Faculté des Sciences, Université Libre de Bruxelles (ULB), Brussels, Belgium; 3 Department of Computer Sciences and Applied Mathematics, Weizmann Institute of Science, Rehovot, Israel; 4 Division of Maternal Fetal Medicine, Helen Schneider Women’s Hospital, Rabin Medical Center, Petah Tikva, Israel; 5 Jesse and Sara Lea Shafer Institute for Endocrinology and Diabetes, National Center for Childhood Diabetes, Schneider Children’s Medical Center, Petah Tikva, Israel; 6 Faculty of Medicine and Health Sciences, Tel Aviv University, Tel Aviv, Israel; Institute for Stem Cell Science and Regenerative Medicine, INDIA

## Abstract

Prediabetes, a subclinical state of high glucose, carries a risk of transitioning to diabetes. One cause of prediabetes is insulin resistance, which impairs the ability of insulin to control blood glucose. However, many individuals with high insulin resistance retain normal glucose due to compensation by enhanced insulin secretion by beta cells. Individuals seem to differ in their maximum compensation level, termed beta cell carrying capacity, such that low carrying capacity is associated with a higher risk of prediabetes and diabetes. Carrying capacity has not been quantified using a mathematical model and at present cannot be estimated from measured glucose and insulin levels in patients, unlike insulin resistance and beta cell function which can be estimated using HOMA-IR and HOMA-B formula. Here we present a mathematical model of beta cell compensation and carrying capacity, and develop a new formula called HOMA-C to estimate it from glucose and insulin measurements. HOMA-C estimates the maximal potential beta cell function of an individual, rather than the current beta cell function. It uses prediabetes as a stress and estimates carrying capacity using the gap between secreted insulin and the needed amount of insulin for homeostasis. We test this approach using longitudinal cohorts of prediabetic people, finding 10-fold variation in carrying capacity. Low HOMA-C associates with higher risk of transitioning to diabetes in a one-year follow up. The interpretation of HOMA-C as a carrying capacity relies on a mathematical model and requires further experimental testing. Quantification of beta cell carrying capacity may help to better understand this understudied risk factor for diabetes.

## Author summary

Prediabetes is a common condition of elevated glucose. Many patients with prediabetes transition into type 2 diabetes, and it is difficult to predict who will transition. One major risk factor is thought to be an inability of beta cells to compensate for deterioration of other physiological parameters, especially insulin sensitivity. The limit for this compensation, in terms of beta cell functional mass, is called beta cell carrying capacity, but there is no way currently to estimate a person’s carrying capacity. Here we use mathematical modeling and a prediabetes longitudinal cohort to develop a method to estimate carrying capacity using fasting insulin and glucose lab tests. This method offers a way for researchers to study the underexplored risk factor of low beta cell carrying capacity, with the aim of preventing the transition to type 2 diabetes. We note that further experimental testing is required to understand HOMA-C and its distinction from HOMA-B.

## Introduction

Prediabetes is defined as a state in which fasting glucose is between 5.6mM and 6.9 mM [1,2] with global prevalence estimated at 10–30% [3]. Prediabetes is a subclinical state that can transition to type-2 diabeteswith a yearly conversion rate of 5–10% [1]. Since type-2 diabetes and prediabetes are associated with morbidity and mortality [2], it is important to understand the origin of prediabetes and to determine which individuals are at risk to transition to diabetes.

One of the main causes of prediabetes is high insulin resistance [4,5]. In insulin resistance, insulin is less effective in removing glucose from the blood and suppressing glucose release from the liver. Insulin resistance can increase rapidly within hours to days in conditions such as inflammation and acute stress. In these situations, insulin resistance is modulated by hormones and cytokines, and is thought to be a physiological knob that allocates glucose appropriately [6]. Insulin resistance also rises in obesity due to inflammation and fat accumulation in muscles and liver. Insulin resistance and beta cell function can be estimated for research purposes from glucose and insulin blood tests using mathematical-model-based formulas such as HOMA-IR and HOMA-B and their more recent improvements [7,8].

Importantly, however, chronically high insulin resistance in many situations does not lead to abnormal glucose levels. This includes most people with obesity, who have high insulin resistance but normal glucose levels [5]. The reason for this normoglycemia is that beta cell function can rise to compensate for insulin resistance [5,9–11]. The increase in beta cell function is due to increased insulin secretion per unit beta cell biomass, and increased total beta cell biomass. Increased beta cell mass in humans likely reflects hypertrophy and/or hyperplasia, depending on the physiological context. Current thought is that postnatal mass change is mainly due to hyperplasia [12], whereas increases in β-cell mass with obesity and pregnancy primarily involves hypertrophy [13–15].

The total ability of beta cells to secrete insulin is called the beta cell functional mass, also called beta-cell function in clinical settings. As beta cell functional mass rises, more insulin is secreted, exactly compensating for insulin resistance [16]. People with obesity indeed have increased beta cell functional mass [17] and increased insulin levels [18].

This raises the question of how prediabetes might occur. Why is compensation not enough to counteract insulin resistance and maintain normal glucose levels and thus prevent prediabetes? One current explanation is metabolic variation between individuals [19,20]. More specifically, individuals can differ in the maximal compensation enabled by beta cells (maximal potential insulin secretion capacity), which can be called the beta cell carrying capacity. Recent studies have developed mathematical models for glucose, insulin and beta cell dynamics incorporating the concept of carrying capacity [21,22].

Individuals with low carrying capacity are predicted to be at risk for prediabetes and diabetes, because a given level of insulin resistance would cause higher glucose than in individuals with high carrying capacity. Low carrying capacity is thus suggested to be a risk factor for prediabetes and diabetes.

However, beta cell carrying capacity cannot currently be measured or estimated. Unlike insulin resistance and beta cell function, which have useful research formulas like HOMA-IR and HOMA-B, there is no analogous HOMA-like formula to estimate the carrying capacity in patients. Therefore, we lack quantitative measurements and mathematical characterization of a potential mechanistic factor that can help to understand some of the variation in risk between individuals.

Here we develop a HOMA- like formula for beta cell carrying capacity based on fasting insulin and glucose blood tests, using a mathematical model of beta cell and glucose dynamics. We call this formula HOMA-C. It aims to estimate the maximal potential beta cell function achievable by an individual with prediabetes. We test this model using a longitudinal dataset of unmedicated patients with prediabetes and newly diagnosed diabetes [23,24] under a dietary intervention. We find that carrying capacity varies by a factor of about 10 between individuals. Carrying capacity according to HOMA-C did not vary strongly with age in a cross-sectional analysis. Low carrying capacity during prediabetes is associated with a high risk of transitioning to diabetes in a one-year follow-up, with about a 4-fold higher risk between the top and bottom HOMA-C quartiles.

## Results

### Mathematical model with beta-cell carrying capacity for prediabetes

To develop a minimal model for beta cell carrying capacity, we use as a basis the classical mathematical models for diabetes, exemplified by the HOMA model [7]. We add to the classical model an equation for beta cell growth, based on the model of Topp et al [9]. Following De Gaetano et al [21], we add a limit to the total beta cell function, the *beta cell carrying capacity*.

This carrying capacity in humans is based on a maximum to the total biomass summed over all beta cells in the body. In humans, beta cells are thought not to proliferate significantly after childhood [12], although some studies suggest adult increase in cell numbers. Beta cells increase their size (hypertrophy) to compensate for high glucose levels. However, cells cannot increase their size indefinitely. Recent data suggests that cells that grow to about twice their normal volume begin to resemble senescent cells and secrete proinflammatory signals [25]. Human data suggest that total beta cell mass can double in obesity [17,26–28] and pregnancy [29], [13–15].

The carrying capacity mathematical model includes a term that forces beta cell functional mass growth rate to become zero when mass *B* reaches a carrying capacity *C*.

We begin with a model without carrying capacity that accounts for beta cell compensation, a simplified version of the model developed by Topp et al [9] where we exclude the glucotoxicity term relevant for glucose levels much higher than those seen in prediabetes. The model has three equations, for glucose *G*, insulin *I* and beta cell mass *B*:


$$\begin{array}{c} \frac{dG}{dt}=m_{\text{0}}-\left(S_{i}\cdot I+k\right)\cdot G \end{array}$$ (1)

$$\begin{array}{c} \frac{dI}{dt}=B\cdot f(G)-\gamma \cdot I \end{array}$$ (2)

$$\begin{array}{c} \frac{dB}{dt}=\mu \cdot B\cdot (\frac{G}{G_{\text{0}}}-\text{1}) \end{array}$$ (3)

where *m*0 is a glucose source term that accounts for liver glucose production plus meal intake, *Si* is insulin sensitivity and *k* is the insulin-independent glucose removal rate. Insulin is secreted by beta cells with functional mass *B* (in units of insulin concentration per unit time) and removed at rate *γ*. The regulation of beta-cell insulin secretion by glucose is described by Hill function $f(G)=\frac{G^{\text{2}}}{h^{\text{2}}+G^{\text{2}}}$ with halfway point *h* [30].The beta-cell functional mass changes with growth rate *μ* to achieve the glucose set point $G_{\text{0}}$.

This model accounts for compensation on the timescale of weeks, as beta cell functional mass grows to buffer changes in parameters such as insulin sensitivity *Si* [10].

To develop a model for the carrying capacity, we follow [21,22] and add to the beta cell growth equation a limit for growth rate that is inspired by ecological carrying capacity:


$$\begin{array}{c} \frac{dB}{dt}=\mu \cdot B\cdot (\left(\frac{G}{G_{\text{0}}}\right)\left(\text{1}-\frac{B}{C}\right)-\text{1}) \end{array}$$ (4)

As beta cell functional mass approaches the carrying capacity C, its growth rate slows down, according to the term $\text{1}-\frac{B}{C}$ adapted from carrying capacity in ecology and in cellular replication [31–33]. In this model, there is sufficient compensation when *B* is much smaller than the carrying capacity. However, when *B* approaches carrying capacity *C*, compensation begins to break down. Insulin production becomes less than needed for sufficient compensation. As a result, glucose levels increase.

We can now derive a formula for estimating the carrying capacity C from lab tests. Assuming that beta-cell mass is at steady state, Eq (4) indicates that C = G B/(G-G0). Estimating B using the well-established HOMA-B formula for beta-cell function, HOMA-B = 20 I/(G-3.5) (with G,I in unit of mmol/L, μU/ml respectively), and a nominal glucose setpoint of *Go*=4.8mM, we arrive at a formula for carrying capacity that we call HOMA-C:


$$\begin{array}{c} HOMA-C=\frac{G\cdot HOMA-B}{G-\text{4}.\text{8}} \end{array}$$ (5)

or in terms of insulin and glucose test values (in mM units for G):


$$\begin{array}{c} HOMA-C=\text{2}\text{0}\cdot \frac{G\cdot I}{\left(G-\text{4}.\text{8})(G-\text{3}.\text{5}\right)} \end{array}$$ (6)

Unlike HOMA-B that estimates *current* beta-cell function, HOMA-C estimates the *maximal* beta cell function that an individual can *potentially* achieve, for example if insulin resistance would rise to very high levels.

One might ask how two measurements- fasting insulin and glucose- can give rise to three estimate equations- HOMA-IR, HOMA-B and HOMA-C. The answer is that we use three equations, Eq (1), (2) and (4) at steady-state to derive three relations for IR, Beta and C as a function of insulin I and glucose G. The original HOMA model effectively had only two equations, lacking the B mass equation 4.

Note that this formula should only be used in cases where fasting glucose is sufficiently higher than the healthy fasting glucose setpoint, which we nominally take to be 4.8mM, to avoid small values in the denominator that can increase errors. The 4.8mM glucose setpoint can be adjusted for different populations. For standard lab test error rates (SI) we estimate that a 20% accuracy in HOMA-C requires fasting glucose higher than 5.3 mM. Thus, the formula can be applied to patients with prediabetes or diabetes.

We also note that HOMA-C will not be accurate in periods where beta cell mass changes strongly over time, since we require dB/dt = 0. Thus, the formula should not be used in transient periods of weeks after changes in health or nutrition.

### Carrying capacity varies widely between individuals

We used the mathematical model of Eq (1), (2) and (4) to estimate the carrying capacity using data from a cohort of *n* = 256 unmedicated people with prediabetes [23,24]. Of these, 8.5% had high enough glucose to be considered diabetic (*G* ≥ 6.9 *m M*/*L*). Each participant had 1–4 measurements over one year of both fasting insulin and glucose.

We first considered the tests performed upon enrollment in the study, considering all prediabetic patients at a single time point to examine the variation of their parameters. The distributions of glucose and insulin measurements are broad (Fig 1A). This can be explained by our model with differing values of carrying capacity for each individual (the curves in Fig 1A). Individuals with low carrying capacity fail to maintain the high insulin levels required for glucose homeostasis at high insulin resistance, in contrast to individuals with high carrying capacity.

**Fig 1:**
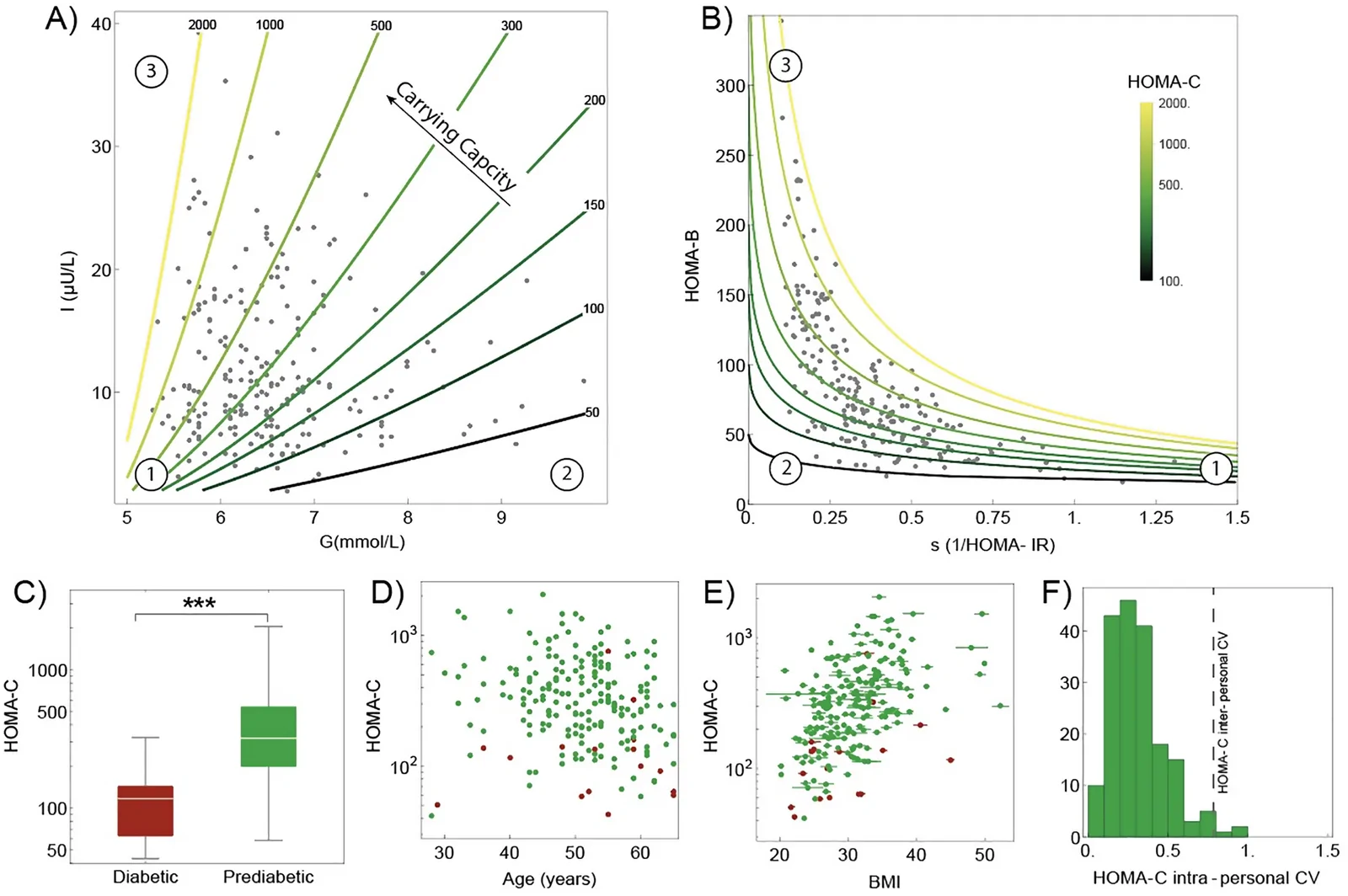
Carrying capacity estimated from insulin and glucose measurements from 256 unmedicated participants with prediabetes from (Ben-Yacov et al., 2021, Rein et al., 2022). (A) Insulin and glucose in the unmedicated prediabetes cohort at enrollment, with outliers removed by excluding data outside of the 90% density contour. Curves denote different values of carrying capacity HOMA-C. (B) Beta cell function (HOMA-B) versus insulin sensitivity (1/HOMA-IR). Curves show different values of carrying capacity HOMA-C. Numbered circles are equivalent points in A and B for orientation. (C) HOMA-C in individuals with prediabetes and diabetes according to the criteria of [Ben-Yacov et al 2021]. (D) HOMA-C versus participant age. Red dots indicate diabetes. (E) HOMA-C versus participant BMI. (F) Distribution of the coefficient of variation (CV) of HOMA-C within an individual’s measurements. Vertical dashed line shows the CV of the mean HOMA-C between individuals. Formulae are HOMA-B = 20 I/(G-3.5), HOMA-IR = I G/22.5 and HOMA-C = G HOMA-B/(G-4.8).

The data can also be visualized in terms of HOMA-IR (a proxy for insulin sensitivity Si) and HOMA-B (a proxy for beta cell function) (Fig 1B). As insulin resistance rises, beta cell function rises and asymptotically reaches the carrying capacity when insulin resistance is very high. Fig 1A shows that the predicted carrying capacity HOMA-C varies by a factor of about 10 between individuals (maximal divided by minimal HOMA-C in the cohort), with a CV of about 75%. This relates to postmortem histology studies that report large variation in total beta cell mass between equal aged individuals [34]. HOMA-B likewise varies between individuals by a factor of 10 (S1A Fig). Note that HOMA-B and HOMA-C are not equivalent – for example HOMA-B of 100 corresponds to individuals with HOMA-C ranging from about 500–1500.

We compared the carrying capacity in the beggining of the trial between individuals with prediabetes and those with diabetes. HOMA-C was 2.5-fold lower on average in those with diabetes (Fig 1C, *p* = 3 10–7). This is in line with postmortem histology studies that showed 50% lower beta cell total mass on average in individuals with diabetes [17].

HOMA-C does not vary significantly with participant age, at least in this cohort in a cross-sectional analysis (Fig 1D). This indicates that age as a risk factor for diabetes might not operate by reducing carrying capacity, but via other mechanisms such as increased insulin resistance, but longitudinal data is required to verify the age dependence of HOMA-C.

Carrying capacity shows mild variation with BMI, rising by a factor of about 2 on average across the range of BMI in the cohort (Fig 1E). This may be due to the cohort composition which includes primarily individuals with prediabetes and not full-blown diabetes. Individuals with high BMI tend to have high insulin resistance and thus transition to diabetes rather than remain prediabetic unless they have a high carrying capacity.

Repeating this analysis using all timepoints in the dataset shows essentially the same conclusions (S2 Fig). We further analyzed a larger dataset containing 20 years of blood tests (7624 glucose and insulin measurements from Centers for Disease Control and Prevention, National Health and Nutrition Examination Survey [35]). HOMA-C was found to be almost 3-fold larger in the prediabetic group than in the diabetic group. It varied weakly with age (cross-sectional analysis) and only moderately with BMI (S3 Fig)

We next asked how the predicted carrying capacity changes in each individual over time. We computed the variation of HOMA-C between the longitudinal measurements of each individual over a year (Fig 1F). This intrapersonal variation is about 25% on average, as quantified by the coefficient of variation (CV = standard deviation/mean). In contrast, HOMA-C varies between individuals by about CV = 75% (Fig 1F, vertical line). Thus, carrying capacity in this dataset may be regarded as a relatively constant trait of a single individual, varying over time by about one third of the inter-person variation, although a low intrapersonal-to-interpersonal CV ratio does not by itself establish that carrying capacity is a fixed physiological trait. Other measures such as HOMA-B, S and DI show an inter-to-intrapersonal variation ratio of ~35%, 50% and 70% respectfully (S1B Fig), which are somewhat wider relative to HOMA-C, except for HOMA-B which varies in a similar magnitude. We show individual trajectories of HOMA-C, glucose, HOMA-B, HOMA-DI and HOMA-S in S4 Fig, where the CVs are presented again after screening for high fasting glucose.

We also tested how quickly insulin resistance rises in this cohort. We evaluated the timescale of insulin resistance (HOMA-IR) variation in the prediabetes cohort [23,24] over a year and find a typical timescale of about 362±41 days. Thus, IR may be considered relatively constant over months (S5 Fig).

This supports the understanding of prediabetes through the model - carrying capacity does not change much over time; instead, over years insulin resistance slowly rises. Beta cell functional mass rises to compensate but comes eventually approaches the carrying capacity, and thus insulin production is insufficient to compensate for the insulin resistance. Glucose levels rise gradually until prediabetes and then T2D thresholds are crossed.

### HOMA-C associates with risk of transition to diabetes more strongly than other insulin-dependent Homa indicators

To test whether low HOMA-C is a risk factor for transitioning from prediabetes to diabetes in this cohort, we considered participants with prediabetes (G < 6.9mM) at enrollment, evaluated their HOMA-C at enrollment, and asked which participants transition to diabetes over the year of follow-up. We focused this calculation on participants who are prediabetic (G > 5.6mM) and who are not on the verge of diabetes (G < 6.6mM). This was done since for the edges (nearly diabetic or not even prediabetic), we expect the transition risk within 1 year to depend mostly on their initial state and fasting glucose than on other parameters such as HOMA-C.

There were 66 participants with at least one follow-up glucose test, and the criterion for transition was a glucose test above 6.9mM. A total of 44 participants transitioned to diabetes. We analyze the risk of diabetes in each quartile of HOMA-C. The top quartile had about a 4-fold lower risk of transition to diabetes than the top quartile, meaning that 4-times more participants transitioned to diabetes in the lower quartile of HOMA-C than in the upper one.

We repeated these tests with other diabetes measures based on lab tests- HOMA-B, HOMA-S, the disposition index [36] HOMA-DI=HOMA-B * HOMA-S, and fasting glucose G. HOMA-C outperforms the insulin and glucose dependent indicators, HOMA-B and HOMA-S, and is slightly less predictive than the only-glucose dependent measures DI and G. Table 1 presents a bootstrapping assessment of the mean risk factor per ratio of each parameter, as defined in Materials and Methods This parameter was chosen to examine the dependence of the risk on the HOMA parameters since there are not many subjects in the cohort and yet we can coarsely quantify the risk dependence on parameters. The full results of the bootstrapping per quartile are shown in Fig 2.

**Table 1:** A bootstrapping assessment of the mean risk factor per ratio of each parameter, defined as $bootstrap\_mean\{mean\{\frac{risk_{q1}}{risk_{q2}},\frac{risk_{q2}}{risk_{q3}},\frac{risk_{q3}}{risk_{q4}}\}\}$, where $q_{i}$ are the quartiles The mean and std were calculated for 1000 bootstraps.

Parameter	Mean Risk/Quartile
G	2.11±0.83
HOMA-DI	2.11±0.83
HOMA-C	1.93±0.66
HOMA-B	1.40±0.38
HOMA-S	1.06±0.27

**Fig 2:**
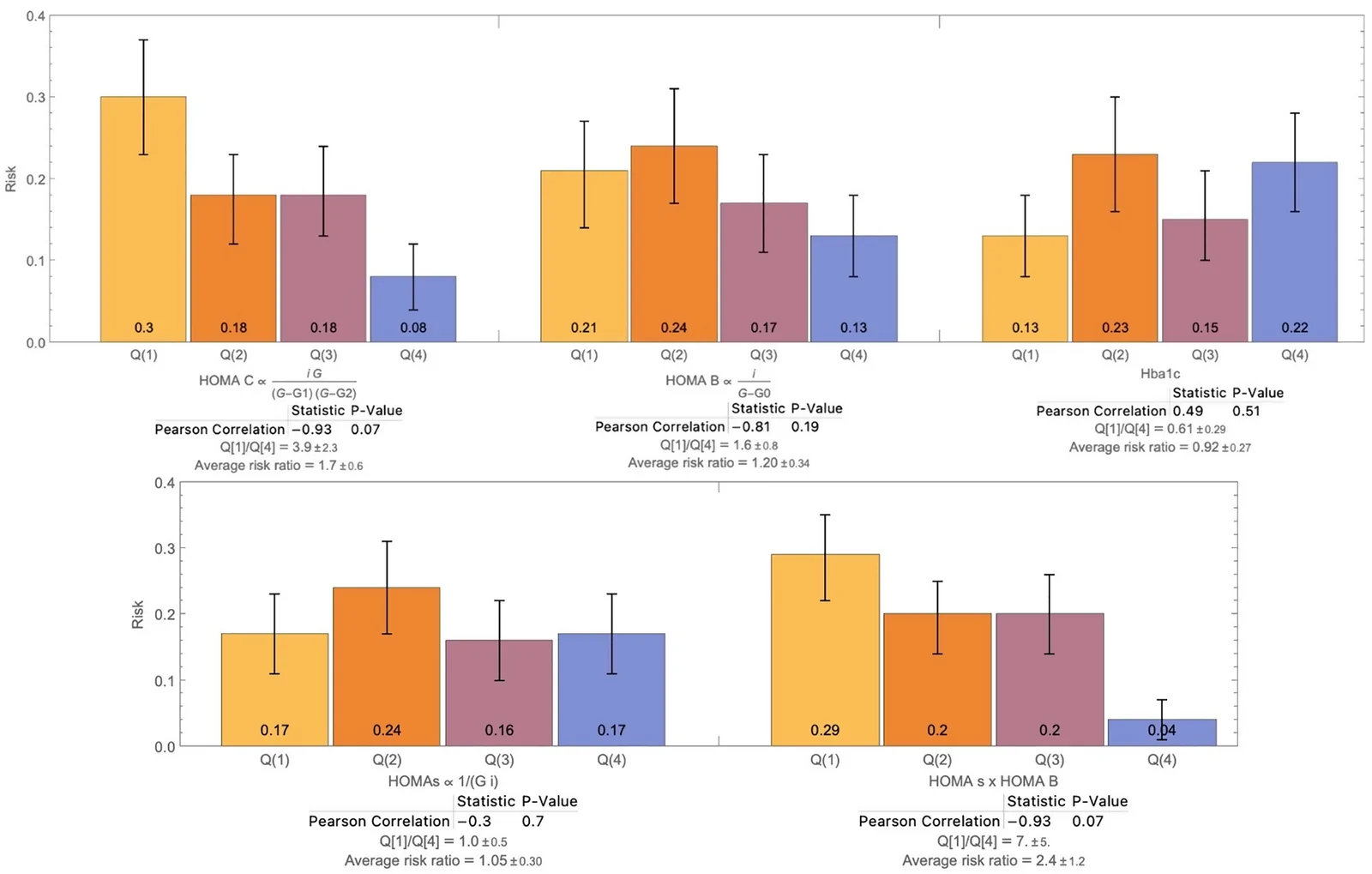
Correlation between diabetes transition risk and 4 diabetes related parameters – HOMA-C, HOMA-B, HbA1C, HOMA-S. The bars show, for each parameter, the frequency of transitioning to diabetes in each quartile of the parameter, e.g., the subject in the lowest quartile of HOMA-C had ~ 30% transition rate while subjects in the highest HOMA-C quartile had only ~8%. The mean risks and confidence intervals were calculated using bootstrapping. The bars are colored by quartile order. Beneath each graph are the Pearson correlation values, the risk ratio between the highest and lowest quartiles, and the average risk ratio between quartiles [q1/q2, q2/q3, q3/q4]. In this cohort, HOMA-C was the most correlated with diabetes risk among the insulin-dependent HOMA indicators HOMA-B and HOMA-S. It was similarly but slightly less correlated than only fasting glucose dependent indicators – G and disposition index HOMA-S X HOMA-B.

We conclude that low HOMA-C estimates for carrying capacity in prediabetes predict transition to diabetes in a one-year follow-up. This is consistent with the notion that low carrying capacity reduces the ability of beta cells to compensate, though, as noted in the Discussion, this predictive association does not by itself distinguish whether HOMA-C captures information beyond a reparameterization of fasting glucose and insulin.

### Integrated model for prediabetes and diabetes subtypes

The carrying capacity model provides a minimal, integrated model of beta cell function and glucose levels that describes the transitions between normoglycemic, prediabetic and diabetic conditions. In addition, it mathematically describes several diabetes subtypes.

To provide intuition, we use a graphic approach to understand the predictions of the model. This graphic approach, called nullcline analysis, is used in the study of dynamical systems [37]. The dynamics described in this analysis include the assumption that insulin -dynamics (minutes) are much faster than Glucose dynamics (hours), as discussed in Materials and Methods. The nullclines each describe the steady state of one parameter if you clamp the other (Fig 3A). One nullcline describes the decrease in steady-state glucose due to beta cell function: the higher beta cell function the lower steady-state glucose, due to the effects of insulin. This is called the *G*-nullcline. The other arm is the increase in steady-state beta cell functional mass *B* due to glucose, which underlies compensation. This is called the *B*-nullcline. The carrying capacity is seen in the shape of the *B* -nullcline, which saturates at a maximum value equal to the carrying capacity. These two nullclines cross at a point which defines the system’s fixed point, because it is the point where both *G* and *B* are at steady state. The fixed point determines the steady-state glucose and beta cell function mass.

**Fig 3:**
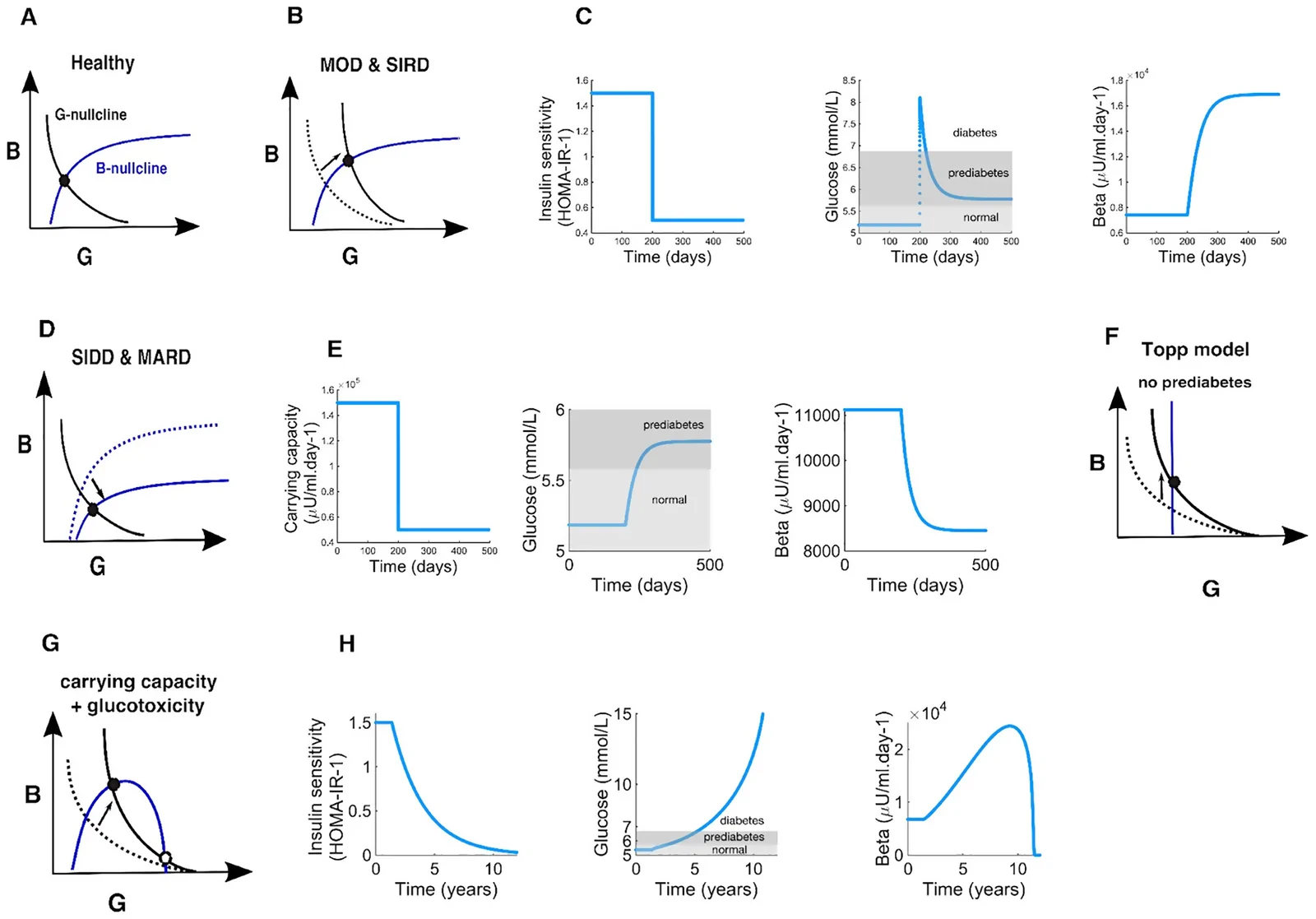
The carrying capacity model provides a framework to understand changes in steady state glucose and beta cell functional mass. (A) Nullclines for the carrying capacity model. (B) Effects of raising the G nullcline as in insulin resistance. MOD - mild obesity-related diabetes, SIRD- severe insulin resistance diabetes. (C) Dynamics of the carrying capacity model for a step-like decrease in insulin sensitivity from 1.5 to 0.5 $HOMA-{IR}^{-\text{1}}$, an arbitrary magnitude to qualitatively reproduce an effect similar to the step-change the original Topp model. A key feature of the carrying capacity modelis that glucose steady state changes when changing SI – only partial compensation occurs, resulting a prediabetic state instead of a return to normoglycemia. The three panels show insulin sensitivity, glucose and beta cell functiona mass in the model. (D) Effects of lowering the B-nullcline as a result of a reduction in beta cell number or health. SIDD- severe insulin deificiency diabetes, MARD- mild age-related diabetes. (E) Dynamics of the carrying capacity model for a step-like decrease in the carrying capacity from $\text{1}.\text{5}{\text{1}\text{0}}^{\text{5}}$ to $\text{5}\cdot {\text{1}\text{0}}^{\text{4}}\frac{\mu UI}{ml\cdot day}$. Fig 3. (F) Nullclines for the linearized Topp model without glucotoxicity. (G) Nullclines for the carrying capacity model with glucotoxicity. A third high glucose fixed point is not shown. (H) The dynamics of the glucotoxicity model (eq. 16) for a slow exponential increase in insulin resistance Si=1.5exp(−αt) with$\alpha =\text{5}\cdot {\text{1}\text{0}}^{-\text{4}}day$. Kinetic parameters: $m_{\text{0}}=\frac{\text{4}\text{8}mmol}{Lday^{-\text{1}}}$, $k=\text{1}.\text{4}\text{4}day^{-\text{1}}$, $\gamma =\text{4}\text{3}\text{2}day^{-\text{1}}$, $h=\text{7}.\text{9}\frac{mmol}{L}$, $C={\text{1}\text{0}}^{\text{5}}{\text{1}\text{0}}^{\text{4}}\frac{\mu UI}{ml}.day$, $G_{\text{0}}=\text{4}.\text{8}\frac{mmol}{L}$ and, $G_{\text{1}}=\text{1}\text{0}\frac{mmol}{L}$. B dynamics are given by Eq. 16.

Physiological conditions and medical interventions can change these nullclines, thus shifting the fixed point and hence the steady-state value of glucose and beta cell functional mass. Some insulin conditions affect the *G*-nullcline, namely the steady state glucose at a given beta cell mass (Fig 3B). This includes insulin resistance, which shifts the nullcline to higher levels of glucose, due to the reduced effectiveness of insulin. This causes a rise in both steady state glucose and beta cell functional mass. Other conditions that affect the *G*-nullcline are excess glucose production by the liver, excess insulin removal rate, reduced insulin-independent glucose removal, and impairments in the beta-cell response to glucose. All of these conditions in the model show a rise in beta cell mass together with a rise in glucose. For example, upon a step-like rise in insulin resistance, the model shows how glucose rises within hours. As beta cell mass increases over weeks, fasting glucose slowly declines, but there is only partial beta-cell compensation in the carrying capacity model, such that fasting glucose declines to a higher value than before, producing a prediabetic stable state (Fig 3C).

In contrast, other conditions affect the *B*-nullcline. This includes a reduction in beta cell health, so that a given glucose level results in lower beta cell functional mass pushing the *B*-nullcline to lower values (Fig 3D). Such changes also shift the fixed point. They give rise to higher glucose levels, but lower beta cell function mass. A similar effect is caused by conditions that lower the beta cell carrying capacity.

Thus changes in the *G*-nullcline should show increases in the steady state values of both *G* and *B*, whereas changes in the *B*-nullcline should show an increase of *G* but a decline of *B*. Such a drop in beta cell secretion capability causes a rapid rise in glucose followed by a decline as beta cell mass grows to compensate (Fig 3C).

Using the distinction between the two nullclines, one can analyze different subtypes of diabetes. Recently, four prevalent subtypes of non-autoimmune diabetes were proposed [38]. Two of these, mild obesity related diabetes (MOD) and severe insulin resistance diabetes (SIRD), are accompanied by a rise of insulin resistance, corresponding to an increase in the *G*-nullcline. Indeed, these types show a rise in both *G* and beta cell function *B* (Fig 3B).

The other two types, severe insulin deficient diabetes (SIDD) and moderate age-related diabetes (MARD) have the opposite phenotype- a rise in glucose but a decline in beta cell functional mass [38]. We propose that these two types of diabetes involve a shift in the *B*-nullcline, compromising beta cell compensation rather than insulin resistance as a primary etiology (Fig 3D).

A similar analysis can be made for mature onset diabetes of the young (MODY), which accounts for about 5% of diabetes cases. Most MODY cases are due to two major genetic variations. The HNF1 variants (50% of MODY) impair insulin secretion, lowering the *B*-nullcline and indeed show the predicted reduced B-cell function along with high glucose [39]. The other major variant, GCK (30%), impairs glucose sensing in beta cells. It thus should affect both nullclines (see nullcline equations in Methods). It has higher beta-cell function than HNF1 (3-fold) and non-MODY T2D (1.5 fold) [39].

The linearized Topp model without carrying capacity or glucotoxicity has different nullclines that cannot capture these subtypes of diabetes (Fig 3F). The Topp model has the same *G*-nullcline as the present model, but the *B*-nullcline is a vertical line at *G* = *G*0. At steady state, compensation always brings glucose back to baseline. The present model differs from the Topp model by having a carrying capacity, and this is the origin of the curved shape of the *B*-nullcline and the existence of prediabetes steady states with glucose above baseline (Fig 3B and 3D).

Finally, we added glucotoxicity to the carrying-capacity model (see SI). Glucotoxicity is an effect, characterized primarily in rodents, in which beta cells are removed or become dysfunctional when glucose reaches very high levels [9,20,40,41]. Glucotoxicity causes the B-nullcline to have a rising and falling shape, since beta cell mass shrinks at high glucose levels (Fig 3G). The two nullclines now cross at three points: a stable fixed point at normal glucose levels and another a stable fixed point at very high glucose in which beta cells mass is zero. Between these is an unstable fixed point.

Rising insulin resistance pushes the *G*-nullcline up, and moves the low stable fixed point and the unstable fixed points closer to each other (Fig 3G). Glucose baseline thus rises as in prediabetes and into the range of type-2 diabetes. At a critical level of insulin resistance the system goes through a saddle-node bifurcation at which the two fixed points collide and annihilate, leaving the high glucose fixed point as the only steady-state solution. Such a process may correspond to a transition to insulin-dependent diabetes at very high insulin resistance (Fig 3H).

## Discussion

We present a HOMA-C formula to estimate the beta-cell carrying capacity - the predicted maximal beta cell function achievable by an indidivual- based on glucose and insulin blood tests. Using a cohort of unmedicated people with prediabetes, we found that inferred carrying capacity varies by a factor of 10 between individuals but varies much less over time in each individual. The lowest quartile of carrying capacity was associated with a 4-fold higher risk of developing diabetes within a year compared to the top quartile.

Carrying capacity for beta cell compensation requires further research. It would be important to study what determines the beta-cell carrying capacity, and how it differs between individuals and across physiological conditions. A recent review summarizes evidence that total beta cell mass and function is lower in prediabetes and in T2D patients on the order of 20–50% compared to healthy individuals [17]. In addition, carrying capacity may be affected by conditions that modulate maximal insulin secretion per beta cell unit mass. These include incretin hormones like GLP1, neuronal inputs to beta cells and drugs like sulfourea, all of which increase beta cell insulin release, and somatostatin secreted by delta cells which reduces insulin release.

HOMA -C appears to be more stable within a person (S1B Fig) than glucose or HOMA-DI in our cohort. Both HOMA-C and HOMA-B appear to be stable in the sense that their intrapersonal CV is about a third of their interpersonal CV (although low interpersonal versus intrapersonal CV is not by itself proof of temporal stability). This contrasts with other indices such as insulin resistance and disposition index where the interpersonal and intrapersonal CV are 0.4 and 0.9 respectively. Further work can test whether HOMA -C might be a strong predictor for prediabetic patients on longer timescales of 5 or 10 years.

One crucial difference between HOMA-B and HOMA-C is that HOMA-C is undefined at euglycemic conditions, G < 4.8mM, whereas HOMA-B is well defined. The reason is that HOMA-C depends on an out-of-homeostasis condition, namely prediabetes, to ascertain maximal beta cell capacity. It is built on the idea of a gap between insulin produced and insulin that would have been enough to return to homeostasis – prediabetes acts as a kind of stress to test the system and reveal its carrying capacity, which is hidden when the system is able to compensate and reach homeostasis. In this way, although it is based on state-dependent variables and not on a dynamic stress test like OGTT, we believe that HOMA-C carries information about carrying (future, maximal) capacity, in contrast to the current capacity measured by HOMA-B. Our main reason for believing this is the mathematical model presented in the paper.

HOMA-C can only be used for individuals with glucose above the normal range (we recommend glucose above 6mM) and in conditions where beta cell functional mass can be assumed to be close to steady state. It should not be used proximally to conditions that cause abrupt changes in insulin resistance such as infections, prolonged illness, or pregnancy, because beta cell functional mass compensation has not reached steady state.

Carrying capacity might also be important when insulin resistance rises at a time when other physiological conditions already call for increased beta cell functional mass. For example, during pregnancy, beta-cell insulin secretion increases to compensate for pregnancy-induced insulin resistance. An additional rise in insulin resistance due to, say, chronic stress or obesity, would be especially harmful because beta cells are already pushed closer to their carrying capacity by pregnancy. This may explain part of the risk for gestational diabetes.

The concept of a carrying capacity also plays a role in adipocyte fat storage. Subcutaneous adipocytes store fat, but their numbers do not change appreciably in adults [42]. They grow in size as more fat is stored. This means that subcutaneous fat also has a carrying capacity. Once this is approached, fat overflow is stored in inner organs as visceral fat, such as in liver, pancreas and muscle. This visceral fat triggers insulin resistance and inflammation [22], and in turn drives prediabetes and diabetes. Populations with low subcutaneous fat carrying capacity are thus predicted to have faster onset of diabetes. This may relate to certain south Asian populations [43]. In addition, ref [44]. points that for some Koreans in their study, T2D and prediabetes can develop even if insulin resistance is slow and modest in degree, as predicted for people with low carrying capacity.

The concept of carrying capacity for an endocrine gland has recently been used in a mathematical model of another subclinical endocrinological condition- subclinical hypothyroidism. Kohanim et al. showed that the carrying capacity of the pituitary cells that secrete thyroid stimulating hormone (TSH) can explain subclinical hypothyroidism in a way that is similar to the present mechanism for prediabetes [32]. Similar carrying capacity effects might be at play in other hormonal systems.

In the present cohort, insulin resistance changed on the timescale of about one year. This is consistent with findings from recently diagnosed patients with T2D from the IMI-DIRECT study [45], in which insulin sensitivity decreased by a factor of about 2 over 36 months in a subpopulation of fast progressors, and showed no change on average in the remainder 90% of the population. Similarly slow rates of change of HOMA-IR in individuals with risk of type-2 diabetes were documented in the Botnia study [45] and other longitudinal studies [46]. Once glucose crosses about 6mM, glucose often rises more rapidly over several years until diabetes occurs [46].

To explain prediabetes under slow rise of insulin resistance (IR), slower than beta cells can compensate, models need to have a limit to beta cell capacity. The present model has a carrying capacity term. Several previous models used an insulin-based inhibition of beta cell function [21,47,48] or a pairwise interaction term with a FFA inflammation term [22]. Without the constraint on the timescales of IR rise, prediabetes arises in additional models [9,49] as shown in S5 Fig. We note that a slow rise in IR is not essential in the present model for prediabetes.

Mathematical models have been important in clinical research on diabetes [7,46,50–53]. The present carrying capacity model and HOMA-C formula which can be estimated from glucose and insulin lab tests may thus help form a more accurate understanding of prediabetes and its transition to diabetes in each individual. Since it is based on simple lab tests, HOMA-C or related approaches might be considered for screening for prediabetes patients at risk to transition to diabetes. HOMA-C differs from HOMA-B in applying only to the prediabetes or diabetes state. Its interpretation is based on a mathematical model and hence requires experimental testing. Different predictive models based on dynamic responses to glucose challenges, such as OGTTs or mixed-meal tests, might ultimately provide better risk estimates; however, the data analyzed here did not include such tests, and fasting measures remain preferable for reasons of cost, simplicity, and patient burden. We hope that this study contributes to further research on beta cell carrying capacity, an understudied risk factor for diabetes.

## Materials and methods

### Carrying capacity model

The Topp model [9] describes the rates of change of blood glucose G, insulin I, and beta cell functional mass B, adapted here as follows:


$$\begin{array}{c} \frac{dG}{dt}=m_{\text{0}}-\left(S_{i}\cdot I+k\right)\cdot G \end{array}$$ (7)

$$\begin{array}{c} \frac{dI}{dt}=B\cdot f(G)-\gamma \cdot I \end{array}$$ (8)

$$\begin{array}{c} \frac{dB}{dt}=\mu \cdot B\cdot (\frac{G}{G_{\text{0}}}-\text{1}) \end{array}$$ (9)

where $m_{\text{0}}$ is liver and postprandial glucose input, $S_{i}$ is insulin sensitivity, k is the insulin-independent glucose removal rate. Insulin is secreted by beta cells with functional mass B (in units of insulin concentration per unit time) and removed at rate

γ. The regulation function of insulin by glucose is a sigmoidal function $f(G)=\frac{G^{\text{2}}}{h^{\text{2}}+G^{\text{2}}}$ with halfway point h. The beta cell functional mass changes with growth rate μ to reach the glucose set point $G_{\text{0}}$.

We ignored the glucotoxicity term at very high glucose levels in the original model $\frac{dB}{dt}=B(\frac{G}{G_{\text{0}}}-\text{1})(\text{1}-\frac{G}{G\text{1}})$. Ignoring this $\left(\text{1}-\frac{G}{G\text{1}}\right)$ term does not change the conclusions of this study, because the glucose levels in the present study are well below the glucotoxicity threshold of $G_{\text{1}}=\text{2}\text{3}$ mmol/L in Topp et al. [9].

This system can be simplified to a set of 2 ordinary differential equations (ODEs) by means of separation of timescales. Insulin is removed with a half-life of about 5 minutes which is faster than glucose removal on the scale of hours and beta cell functional mass growth on the scale of weeks. Insulin thus equilibrates rapidly compared to the slow timescale, so that we use a quasi-steady state assumption with $\frac{dI}{dt}=\text{0}$. The quasi-steady-state solution is$I_{qss}=Bf(G)\cdot \gamma^{-\text{1}}$. Substituting this expression for *I* in the equation for glucose reduces the system to two equations:


$$\begin{array}{c} \frac{dG}{dt}=m_{\text{0}}-\left(S_{i}\cdot Bf(G)\cdot \gamma^{-\text{1}}+k\right)\cdot G \end{array}$$ (10)

$$\begin{array}{c} \frac{dB}{dt}=\mu \cdot B\cdot (\frac{G}{G_{\text{0}}}-\text{1}) \end{array}$$ (11)

We use the kinetic parameter values used by Topp et al. and Karin et al. [9,10].

**Table:** 

Parameter	Value	Units
$m_{\text{0}}$	48	mmol/L${day}^{-\text{1}}$
k	1.4	${day}^{-\text{1}}$
γ	432	${day}^{-\text{1}}$
h	7.9	mmol/L

The present model extends the Topp model by introducing a carrying capacity for beta cells, C. Introducing the latter in the 2 ODE version of the Topp model, we obtain


$$\begin{array}{c} \frac{dG}{dt}=m_{\text{0}}-\left(S_{i}\cdot Bf(G)\cdot \gamma^{-\text{1}}+k\right)\cdot G \end{array}$$ (12)

$$\begin{array}{c} \frac{dB}{dt}=\mu \cdot B\cdot (\frac{G}{G_{\text{0}}}(\text{1}-\frac{B}{C})-\text{1}) \end{array}$$ (13)

where *C* is the carrying capacity.

In the limit of very slow changes in *Si* relative to the compensation time, which is appropriate to the present estimates, one uses $\frac{dG}{dt}=\text{0}$ to obtain the following relation between glucose and insulin sensitivity$S_{i}$


$$\begin{array}{c} S_{i}=\frac{\gamma \cdot (\frac{m_{\text{0}}}{G}-k)}{C\cdot f(G)\left(\text{1}-\frac{G_{\text{0}}}{G}\right)} \end{array}$$ (14)

with $f(G)=\frac{G^{\text{2}}}{h^{\text{2}}+G^{\text{2}}}$

The relation between functional beta cell mass B and $S_{i}$ is


$$\begin{array}{c} S_{i}=\frac{\gamma \cdot (\frac{m_{\text{0}}}{G}(\text{1}-\frac{B}{C})-k)}{h(B)\cdot B} \end{array}$$

with $h(B)=\frac{G_{\text{0}}^{\text{2}}}{G_{\text{0}}^{\text{2}}+h^{\text{2}}{\left(\text{1}-\frac{B}{C}\right)}^{\text{2}}}$

Based on this model, we define the HOMA-C formula based on the steady state assumption for *B*, namely *dB/dt = 0*. This provides $C=\frac{G\cdot B}{G-G_{\text{0}}}$. Since B is widely estimated using the HOMA-B formula, HOMA-B = 20 I/(G-3.5), we propose the following formula for carrying capacity:


$$\begin{array}{c} HOMA-C=\frac{G}{G-\text{4}.\text{8}}HOMA-B, \end{array}$$

with $G_{\text{0}}$=4.8 mmol/L.

### Data from an unmedicated prediabetic cohort

We analyze data from a cohort described in [23,24]. The cohort consists of unmedicated prediabetic and diabetic adults who underwent a 6-month dietary intervention and additional 6-month follow-up with several (up to four) blood tests of fasting glucose and insulin levels. We only analyzed data from patients with at least one fasting glucose and insulin measure simultaneously, leading to 256 participants (111 women aged 50±8years, 145 men aged 51±7years), 8.5% being diabetic at the beginning of the dietary intervention. Patients were included in the diabetic cohort if either their blood glucose or their HbA1c levels were elevated [24].

Insulin was measured with the use of a chemiluminescent microparticle immunoassay (ARCHITECT insulin assay) with precision of less than 7% CV and plasma glucose was measured with the use of a hexokinase method (GLUC2 assay, cobas; Roche) with precision of about 2% CV.

From these data, we estimate how individual insulin sensitivities and functional beta cell mass evolve over time using the HOMA-IR and HOMA-B indices [7], $HOMA-IR=\frac{G\cdot I}{\text{2}\text{2}.\text{5}}$ and $HOMA-B=\frac{\text{2}\text{0}\cdot I}{G-\text{3}.\text{5}}$ where G and I are fasting glucose in mmol/L and insulin in μU/ml.

As in Topp et al. [9], we defined the rate of change of insulin sensitivity as $\alpha =|\frac{\text{1}}{S_{i}}\frac{dS_{i}}{dt}$|. We calculated the rate of change between each measurement for each patient of the cohort as $\left|\frac{\Delta logS_{i}}{\Delta t}\right|$.

### Data from the National Health and Nutrition Examination Survey (CDC)

We analyze data from the National Health and Nutrition Examination Survey (CDC,32). It consists of blood tests (fasting glucose and insulin) from 1999 to 2018. After filtering out the outliers for glucose, insulin and BMI, we obtained 7624 data points in the prediabetic (83%) and diabetic range (those who reported taking insulin or glucose lowering medications excluded). For prediabetic individuals, mean HOMA-C = 640.5 + /- 5.6 (mean + /- SEM) and for diabetic ones, mean HOMA-C = 215.7 + /- 5. Whether HOMA-C is predictive of the risk to transition from prediabetes to diabetes could not be tested with this cross-sectional dataset.

### Carrying capacity model nullclines

We compute the nullclines of the system, that is the curves corresponding to $\frac{dG}{dt}=\text{0}$ and $\frac{dB}{dt}=\text{0}$. The G-nullcline ($\frac{dG}{dt}=\text{0}$) is


$$\begin{array}{c} B=\frac{\gamma }{S_{i}\cdot f(G)}(\frac{m_{\text{0}}}{G}-k) \end{array}$$

Without carrying capacity (that is, C=∞), as in the modified Topp model, the B-nullcline is vertical ($G=G_{\text{0}}$) and there is always compensation. However the presence of a finite carrying capacity C bends the B-nullcline as


$$\begin{array}{c} B=C(\text{1}-\frac{G_{0}}{G}) \end{array}$$

When the value of *Si* decreases, glucose levels remain nearly constant at first because the rise of B cell mass provides compensation. However, when B approaches its carrying capacity C glucose levels start to rise: this is the onset of prediabetes. Note that when insulin resistance becomes very high, that is, in the limit of Si→0, the state of the system converges to $G=\frac{m_{\text{0}}}{k}$ and $B=C(\text{1}-\frac{G_{\text{0}}k}{m_{\text{0}}})$.

### Model with carrying capacity and glucotoxicity

The Topp model originally contains a glucotoxicity term [9]. If we add such a term to our model with carrying capacity (Fig 3G and 3H), we obtain


$$\begin{array}{c} \frac{dG}{dt}=m_{\text{0}}-\left(S_{i}\cdot qBf(G)\cdot \gamma^{-\text{1}}+k\right)\cdot G \end{array}$$ (15)

$$\begin{array}{c} \frac{dB}{dt}=\mu \cdot B\cdot ((\frac{G}{G_{\text{0}}}+\frac{G}{G_{\text{1}}})(\text{1}-\frac{B}{C})-\text{1}-\frac{G^{\text{2}}}{G_{\text{0}}G_{\text{1}}}) \end{array}$$ (16)

When B≪C, that’s when cells are far from carrying capacity, we recover the original Topp et al equation:


$$\begin{array}{c} \frac{dB}{dt}=\mu \cdot B\cdot (\frac{G}{G_{\text{0}}}-\text{1})(\text{1}-\frac{G}{G_{\text{1}}}) \end{array}$$ (17)

### Bootstrapping analysis of diabetes transition risk (Fig 2)

To evaluate how each metabolic parameter relates to the risk of transitioning from prediabetes to diabetes, we performed a non-parametric bootstrapping analysis using fasting glucose and insulin data from all participants with at least two measurements and baseline glucose ≤ 6.9 mM. Individuals who reached glucose > 6.9 mM during follow-up were classified as having transitioned to diabetes.

For each participant, we calculated the parameters HOMA-B = 20 I/ (G − 3.5), HOMA-S = 22.5/ (G I), HOMA-C = G HOMA-B/ (G − 4.8), the disposition index DI = HOMA-B × HOMA-S, and the ratio HOMA-B/ HOMA-C, using the first available fasting test.

To estimate uncertainty in the risk curves shown in Fig 2, the dataset was resampled with replacement 1 000 times. In each resample, participants were divided into quartiles according to each parameter. Within each quartile, we computed the fraction of participants who transitioned to diabetes, representing the empirical transition risk. The mean and standard deviation of this risk across bootstrap iterations were used to plot the bars and error ranges in Fig 2.

For a compact numerical comparison (Table 1), we defined a mean risk-ratio index as the average ratio of successive quartile risks, $Q_{i}$:


$$\begin{array}{c} \left\{mean\{\frac{Q_{\text{1}}}{Q_{\text{2}}},\frac{Q_{\text{2}}}{Q_{\text{3}}},\frac{Q_{\text{3}}}{Q_{\text{4}}}\}\right\} \end{array}$$ (18)

This value was sampled over bootstrapping iterations and its mean and standard deviation are shown in Table 1.

This index summarizes how sharply transition risk decreases across quartiles of each parameter, or increases, if the parameter positively affects transition risk.

## Supporting information

S1 Fig Distribution of different HOMA- parameters within the cohort of 256 unmedicated participants with prediabetes from (Ben-Yacov et al., 2021, Rein et al., 2022). A) Similar to Fig 1A, presenting the distribution of the cohort in the fasting G-I plane, with the addition of the equi-HOMA-C (left) and HOMA-B (right) curves. Red dots indicate subjects who have transitioned to diabetes during the research. B) Distribution of the coefficient of variation (CV) within an individual’s measurements, for HOMA- S, B, DI (disposition index, S*B) and C. The vertical arrow in each histogram shows the CV between individuals. Formulae are HOMA-B = 20 I/(G-3.5), HOMA-IR = I G/22.5 [HOMA-S = 1/HOMA-IR] and HOMA-C = G HOMA-B/(G-4.8).
(TIFF)

S2 Fig Carrying capacity equation HOMA-C for all insulin and glucose measurements from 256 unmedicated participants with prediabetes from (Ben-Yacov et al., 2021, Rein et al., 2022). Error bars show the std of measurements over all timepoints for each individual. This figure is the same as Fig 1, except that all measurements are used and not only the first measurement at enrollment.
(TIFF)

S3 Fig Carrying capacity estimated from insulin and glucose measurements from 7624 unmedicated participants with prediabetes from NHANES (Centers for Disease Control and Prevention, National Health and Nutrition Examination Survey). (A) Insulin and glucose in prediabetic and unmedicated diabetic patients, with outliers removed by excluding data outside of the 95% density contour. Gray contours contain 75%, 50% and 25% of prediabetic patients respectively. Red contours contain 75%, 50% and 25% of diabetic patients respectively. Curves denote different values of carrying capacity HOMA-C. (B) Beta cell function (HOMA-B) versus insulin sensitivity (1/HOMA-IR). Curves show different values of carrying capacity HOMA-C. (C) HOMA-C in individuals with prediabetes and diabetes. (D) HOMA-C versus participant age. (E) HOMA-C versus participant BMI. Formulae are HOMA-B = 20 I/(G-3.5), HOMA-IR = I G/22.5 and HOMA-C = G HOMA-B/(G-4.8).
(TIFF)

S4 Fig Longitudinal trajectories of the cohort throughout the measurement period, for different HOMA- parameters. 109 trajectories were taken, with an initial fasting glucose greater than 6 mmol/L to focus on the change of the parameters during prediabetes and diabetes. For each parameter, the mean intra- and inter-individual CVs, and their ratio, are computed. HOMA-B,C,S are more constant along the year of measurement than DI,G, and HOMA-B,C have a lower intra- to inter- personal variance ratios. (A) Fasting glucose. Intra CV 4.6%, inter CV 6.2%, ratio: 0.74. (B) HOMA-C. Intra CV 21.3%, inter CV 71%, ratio: 0.3. (C) Disposition index. Intra CV 13.5%, inter CV 15.3%, ratio: 0.88. (D) HOMA-S. Intra CV 19.7%, inter CV 50.3%, ratio: 0.39. (E) HOMA-B. Intra CV 17.5%, inter CV 66%, ratio: 0.27.
(TIFF)

S5 FigSlow changes in insulin resistance on the scale of a year estimated from a prediabetic cohort.We analyze the longitudinal data from the cohort of *n* = 256 unmedicated people with prediabetes [23,24].To define the rate of change of *Si* we fit exponential dynamics, *Si* ~ *exp(αt)*, to every two consecutive measurements. Such exponential dynamics were proposed by Topp et al. [9], but any rising dynamics with a characteristic timescale would suffice to assess the rate of change of insulin resistance. We therefore defined the rate of change of insulin sensitivity between every two as $\alpha =\frac{\Delta logS_{i}}{\Delta t}$ where α has units of 1/time. This yields a timescale for changes in insulin resistance of $T_{R}=\text{1}/\alpha$. We plot (A) Dynamics of insulin sensitivity (defined as 1/ HOMA-IR) for 4 patients over time. (B) Histogram of the timescale of changes in insulin resistance $T_{R}$ for all pairs of consecutive measurements in the cohort which shows a broad distribution. The mode is at $T_{R}=\text{3}\text{6}\text{2}\pm \text{4}\text{1}$ days and 95% of the data exceeds 104 days. The timescale is similar for males and females ($T_{R}=\text{3}\text{7}\text{5}\pm \text{6}\text{2}$ days for males, $T_{R}=\text{3}\text{4}\text{0}\pm \text{3}\text{5}$ days for women). The timescale for individuals with diabetes is also similar ($T_{R}=\text{3}\text{4}\text{4}\pm \text{1}\text{0}\text{3}$ days), as it is for upper and lower quartiles of BMI and age ($T_{R}=\text{2}\text{8}\text{2}\pm \text{7}\text{0}$ and $T_{R}=\text{3}\text{4}\text{6}\pm \text{8}\text{3}$ days for BMI; $T_{R}=\text{3}\text{1}\text{4}\pm \text{4}\text{1}$ and $T_{R}=\text{3}\text{4}\text{3}\pm \text{4}\text{1}$ days for age) respectively. We conclude that the timescale of changes in insulin resistance is about one year in the prediabetes cohort.(TIFF)

S6 FigModel with carrying capacity can explain prediabetes when insulin resistance rises slowly, in contrast to a model without carrying capacity.(A) A model with no carrying capacity, the modified Topp model, shows glucose near baseline when the compensation time $T_{comp}$ is 3–6 times slower than the rate of change of insulin resistance $T_{R}$ (dark blue line). TR is timescale ratio. (B) Model presented here with beta cell carrying capacity shows prediabetes at the observed time scale ratio, and in fact at all time scale ratios. Kinetic parameters:$m_{\text{0}}=\frac{\text{4}\text{8}mmol}{Lday}$, $=\text{1}.\text{4}\text{4}day^{-\text{1}}$, $\gamma =\text{4}\text{3}\text{2}day^{-\text{1}}$, $h=\text{7}.\text{9}\frac{mmol}{L}$are the same for both models (Topp et al.,2000), and for the carrying capacity model $C={\text{1}\text{0}}^{\text{5}}{\text{1}\text{0}}^{\text{4}}\frac{\mu UI}{ml}.day$ and $G_{\text{0}}=\text{4}.\text{8}\frac{mmol}{L}$. Simulations used exponentially rising insulin resistance with timescale $T_{R}$ starting from *Si* = 1.5.(TIFF)
