# Supplementary material for: Quantification of beta-cell carrying capacity in prediabetes
Source: PLoS Comput Biol. 2026 Sep 15;22(9):e1014589. doi: 10.1371/journal.pcbi.1014589 (PMC13638054; doi:10.1371/journal.pcbi.1014589)
Supplement: Supplementary file 1 [file pcbi.1014589.s008.pdf]

Dear Dr Woller,

As with all papers reviewed by the journal, your manuscript was reviewed by members of the editorial board and by several independent reviewers. In light of the reviews (below this email), we would like to invite the resubmission of a significantly-revised version that takes into account the reviewers' comments. Additionally, there are several concerns about possibly over-extrapolating the well known model by Topp, as well as what seems like some strawman arguments that have been made. These should be carefully addressed.

We cannot make any decision about publication until we have seen the revised manuscript and your response to the reviewers' comments. Your revised manuscript is also likely to be sent to reviewers for further evaluation.

When you are ready to resubmit, please upload the following:

[1] A letter containing a detailed list of your responses to the review comments and a description of the changes you have made in the manuscript. Please note while forming your response, if your article is accepted, you may have the opportunity to make the peer review history publicly available. The record will include editor decision letters (with reviews) and your responses to reviewer comments. If eligible, we will contact you to opt in or out.

[2] Two versions of the revised manuscript: one with either highlights or tracked changes denoting where the text has been changed; the other a clean version (uploaded as the manuscript file).

Important additional instructions are given below your reviewer comments.

Please prepare and submit your revised manuscript within 60 days. If you anticipate any delay, please let us know the expected resubmission date by replying to this email. Please note that revised manuscripts received after the 60-day due date may require evaluation and peer review similar to newly submitted manuscripts.

Thank you again for your submission. We hope that our editorial process has been constructive so far, and we welcome your feedback at any time. Please don't hesitate to contact us if you have any questions or comments.

Sincerely,

Sunil Laxman, PhD
Academic Editor

PLOS Computational Biology

Jason Haugh
Section Editor
PLOS Computational Biology

****

Reviewer's Responses to Questions

Comments to the Authors:
Please note here if the review is uploaded as an attachment.

Reviewer #1: This paper explores the impact of assuming a limitation in carrying capacity (CC) in beta-cell functional mass. A modified version of the Topp model is used (ref. 9). CC has been incorporated in other models of glucose-insulin homeostasis and diabetes derived from Topp (e.g, cited refs. 17, 18), but as a secondary effect to the main player, glucotoxicity. The present paper shows that CC in the absence of glucotoxicity can lead to prediabetes and diabetes and that a crippled version of Topp without glucose toxicity or CC cannot lead to disease (Fig. 4A). In other words, CC is shown to be sufficient but is not shown to be necessary for disease.

We thanks the reviewers for this endorsement

The new model also assumes that beta-cell compensation to insulin resistance is much faster than insulin resistance itself, which permits reduction of the three-variable system to two dimensions and application of phase-plane analysis. I find the assumption questionable, though they show data that is supportive and some of the conclusions that follow dubious. The authors should at least acknowledge that this assumption is radically different from other models of this class.[NT2]

Response: We now clarify the text which was confusing on that point. We add on line 183: “Reduction of the two variable system is based on assuming that insulin changes to a meal are much faster than the timescale beta cell compensation. We believe this is a plausible assumption at least for beta cell compensation due to gene expression and cell biomass growth. We also note that reduction to two dimensions is not essential for the conclusions, and is done for presentation purposes and to have an analytical formula for HOMA-C (rather than a numerical computation).”

Moreover, we now acknowledge in the text that other models (such as the Topp model) assume that insulin resistance is much faster than beta cell compensation (line 387): “Without the constraint on the timescales [beta cell vs IR], prediabetes rises in additional models (9,52), as demonstrated in Fig. 4.” Thus, insulin resistance outstrips the ability of beta cells to compensate.

In the present model, the situation is different, prediabetes will occur regardless of the relation between these timescales- either fast or slow insulin resistance will cause prediabetes once the carrying capacity is approached. This is similar in spirit to certain models of prediabetes [Ref 17,18,49,50], which have incorporated the concept of a carrying capacity, either by that name (17) or by a pairwise interaction term which can be interpreted in the same manner (18). The model presented in the present paper is a minimal model version.

Specific Comments:

[C.2]L. 99 - 100, “carrying capacity … does not vary strongly with age”: Most of the data shown for this is cross-sectional, and the small set of longitudinal data only covers one year. It seems unlikely that a quantity based on fasting insulin and glucose would not change markedly over longer time periods. This statement needs to be qualified.[NT3]

Response: Changed to (line 106) "We find that carrying capacity varies between individuals by a factor of about 10. Carrying capacity according to HOMA-C did not vary strongly with age in a cross-sectional analysis."

L 127 Eq. 3: the linearized Topp model predicts exponential growth without limit. Linearization is justified by saying that the increases in glucose during pre diabetes are well below the threshold where positive feedback leading to diabetes is set in Topp (L. 510). The Topp model is poorly calibrated, but other models have shown that glucotoxicity is by itself sufficient for prediabetes.[NT4]

Response: We agree and have now made our text more accurate. The models we are aware of to explain prediabetes have either a carrying capacity or another way to limit beta cell function, such as the Topp-IR model (17), either, as we show in Fig.4, give rise to prediabetes when beta-cell compensation is slow enough, which is true for for (52), where they specifically state in Fig.6 “The only difference between the 2 cases is the rate at which SI falls”, referring to what determines transition to diabetes in their simulations. We added (line 387): “Without the constraint on the timescales [beta cell vs IR], prediabetes rises in additional models (9,52), as demonstrated in Fig. 4.”

L. 235 - 245: Carrying capacity is shown to predict transition from prediabetes to diabetes. However, the curves of constant CC in Fig. 1B are very similar to curves of constant disposition index defined by HOMA IS * HOMA B. Disposition indices based on various measures of insulin sensitivity and beta-cell function are good detectors and predictors of diabetes, and I believe that the favorable properties shown for HOMA C may arise from its similarity to a disposition index. To be convinced otherwise, I would need to see evidence that HOMA C is a better predictor than the HOMA disposition index or HOMA B, which it also closely resembles.[NT5]

Response: We have now evaluated HOMA-B and disposition index = HOMA-IS * HOMA-B. We compared their ability to predict onset of diabetes over the year of followup in our cohort. Bootstrapping to evaluate the risk dependence for different parameters, we find that HOMA-C is similar or slightly less predictive than only-glucose dependent measures (all of which are equivalent in their predictions), and outperforms measures which also depend on insulin. We now add a table to the text summarizing the mean risk factor per quartile (line 282).

Parameter	Mean Risk/Quartile
G	2.13+-1.2
HOMA-DI	2.13+-1.2
HOMA-B/HOMA-C	2.13+-1.2
HOMA-C	2.08+-1.1
HOMA-B	1.3+-0.35
HOMA-S	1.02+-0.4

We add in the discussion (line 409): “We find that the risk of transitioning to T2D within a year reduces by about 2.1 per quartile of HOMA-C, and only by 1.4 per quartile of HOMA-B. At least in the cohort analyzed in this paper, HOMA-C was a more significant predictor for diabetes than other insulin-dependent HOMA indicators, HOMA-B and HOMA-S. It was similar to only-glucose dependent indicators: fasting G and a HOMA version of the disposition index HOMA-DI= HOMA-B /HOMA-IR. The purpose of the present study is to provide an estimate of the beta cell carrying capacity and not to predict diabetes, a task better carried out by machine learning approaches using more complete information about the patient (58,59,60). HOMA-C may serve as one of the inputs to such prediction algorithms to improve their performance.”

L. 280 - 294, postpartum recovery of glucose: This is one of the data sets used to justify that beta-cell compensation is much faster than insulin resistance. The change in glucose is only 0.14 mM, about 3%. I question whether such small changes shed meaningful light on the much bigger changes as people progress to prediabetes.

Response: We have now removed the part of the paper that deals with timescales due to this feedback, both postpartum and seasonal data.

L. 323 - 4 and Fig. 4A, “the classic Topp model … can’t explain prediabetes: What is shown is that the linearized Topp model is unable to explain prediabetes. This is trivially true, since functional mass can grow without bound. Even in the Topp model with glucotoxicity (used in Fig. 5G), the predicted increase in mass is implausibly large, but other better calibrated models with glucotoxicity but without CC can account for prediabetes, for example Fig. 7 in Ref. 9 [NT7] and Fig. 2 in https://pubmed.ncbi.nlm.nih.gov/32663101[NT8]. The same applies to L 431 - 432 in the Discussion.[NT9]

Response: We thank the reviewer for this comment that helped us be more accurate. We agree that the model of Ref 9 and the new reference we have now added show prediabetes without carrying capacity. These models assume a beta-cell growth rate on the scale of several years, slower than the rise of insulin resistance, which indeed shows prediabetes without CC. We removed L 431-432, and added this reservation to the text (line 384):

“To explain prediabetes under the conditions where beta-cell growth is faster or similar to the rate at which insulin resistance rises, models need to have a limit to beta cell capacity. The present model has a carrying capacity term. Several previous models used an insulin-based inhibition of beta cell function (17,49,50) or by a pairwise

interaction term with a FFA inflammation term (18). Without the constraint on the timescales, prediabetes rises in additional models (9,52)."

L. 369 and Fig. 5C: the response to a step-wise reduction in Si is shown to result in prediabetes. However, prediabetes results from a slow decline in Si over time, not a step. Topp-like models predict that after a step, glucose will come back to baseline. That seems like the right prediction, but I don't know of any data to settle the question. More importantly, as stated above, when Si declines slowly, the Topp model and its successors can exhibit prediabetes, so the example of Fig. 5C does not refute Topp-like models or demonstrate the necessity of CC.

Response: Necessary comment which helped us clarify the text. The step-like decrease is only a heuristic description which does not give concise evidence. In Fig. 3 we bring a slow decline of Si. We have now modified the text of this figure: "A step like-decrease is a heuristic and over simplified input for diabetes, where the more realistic input is the slow decline in Fig. 3".

L 414 and Fig. 5G: While the reduced Topp model with carrying capacity and insulin set to steady state shows a saddle-node bifurcation, in the original Topp model, with glucotoxicity but no CC, the transition to diabetes is not through an SN. See https://pubmed.ncbi.nlm.nih.gov/26300065/. The transition is more complicated. This casts doubt on the validity of the phase plane analysis.

Response: We have now added this caveat and reference (line 348) "We note that the original TOPP model transitioned to diabetes through a saddle-node bifurcation (50). The present model transitions to prediabetes by a continuous process of approach to carrying capacity. Thus, for phase plane analysis to be valid, the rate of insulin resistance change must be slow compared to other timescales."

[NT12]

L. 439 - 440: "Insulin sensitivity decreased by a factor of about 2 over 36 months": This is much slower than the time scale in Figure 2. Please comment.

Response: The quote is from another paper, Bizzoto et al 2021 (ref 43) which studied a different cohort with potentially different characteristics. We believe that the main difference is the comparison between the common value in our analysis, to the average value in ref 43. In figure S4, in our cohort, the most common (mode) timescale is around ~14 months. The average timescale, however, is higher - 43 months when removing outliers. This is quite similar to the reference paper. Our assessment of this timescale, which is based on 1 year of tracking, has a greater variance than that of the reference

paper, but they both present similar mean values for T-IR. We add in the text (line 378): “The average timescale in our cohort is 43 months when removing outliers, similar to this reference paper.”

L. 512: Setting insulin to steady state in the phase plane analysis of Fig. 5 is justified by the clearance rates of insulin and glucose. But if insulin is really faster than glucose, why does it lag behind glucose in glucose tolerance tests?

Response: This effect may be due to the measurement in the peripheral compartment (serum insulin), in which insulin has a longer half-life. In addition, this assumption does not change the rise of prediabetes and diabetes in the carrying capacity model, and was used to get an analytic formula for HOMA-C.

Minor Points:

L. 46, “a low number of insulin-producing beta cells, called cell carrying capacity”: this is not correct. CC is a limitation in the ability to expand cell number or functional mass, now low number per se.[NT16]

Response: we have fixed this error, Changed to (line 46): "One major risk factor is thought to be an inability of beta cells to compensate for deterioration of other physiological parameters, especially insulin sensitivity. The limit for this compensation, in terms of beta cell functional mass, is called beta cell carrying capacity, …"

L. 360, “Some I changes”: seems like a typo.

Response: Changed to " Insulin conditions affect the…" (line 308)

L.378, “Fig. 5C”: should be 5D. - FIXED

L. 387, ref. 37: should be 39. - FIXED

L. 393, ref. 38: should be 40. - FIXED

L. 450, “sulforea” is misspelled. - FIXED

L. 462, “reduces by about 1.7 per quartile of HOMA-C”: I don’t understand what this number means. Also, cite the study it is based on.

Response: This point is now better explained in the text, and the risk calculations were changed to a more intuitive parameter. The “risk per quartile” is calculated by the average of the quartiles’ risk ratios, i.e. the average of the ratios between adjacent

histogram bars in Fig. 2. Each bar represents the rate of occurrence of transition to diabetes within the cohort in each quartile of a parameter. This is a result of this paper and not cited from another study, now it is written more clearly in the results, discussion and figures.

Reviewer #2: In a rather clever move the authors extend the Topp model to add a carrying capacity term in the B equation. This allows them to explain prediabetes, which as the authors rightly point out, was not explained in the original Topp model. The authors show this is a useful extension by explaining NHANES data and diabetes subtypes as well. This is a welcome addition to the HOMA literature as well, because the authors are able to introduce a new index HOMA-C to deal with prediabetes.

We thank the reviewer for this endorsement

I must say that I was a somewhat surprised by the timescales that the authors show for changes in beta-cell mass as well as insulin resistance. I also note that the HOMA model used in the paper is the classical, older version; nowadays the iHOMA model(s) is often claimed to be "better".

Response: We now add references to the new version of HOMA. We retain the old and simple version because it helps us reach an analytic formula for HOMA-C.

I would like to ask the authors the following question. There are populations such asin southeast Asia where it is claimed that (i) about 50% of people that become prediabetic will go on to develop overt diabetes, and (ii) typically within three years, say. How would you explain these observations within your framework?

Response: We would speculate that in certain populations insulin resistance rises faster than in others. For example, fat has a limited carrying capacity. Once this is exceeded, excess fat is stored in inner organs as visceral fat, triggering insulin resistance. It is possible that populations with low fat carrying capacity are prone to larger spillover and thus faster rise in insulin resistance. Such a rapid rise in our model would trigger a fast onset of diabetes.

We now add to the discussion (line 448): “The concept of a carrying capacity also plays a role in adipocyte fat storage. Subcutaneous adipocytes store fat, but their numbers do

not change appreciably in the adult (53). However, ref. 53 shows that they stop proliferating by the age of in your 20s. They grow in size as more fat is stored. This means that subcutaneous fat also has a carrying capacity. Once this is approached, fat overflow is stored in inner organs as visceral fat, such as in liver, pancrase and muscle. This visceral fat triggers insulin resistance and inflammation (18), and in turn drives prediabetes and diabetes. Populations with low subcutaneous fat carrying capacity are thus predicted to have faster onset of diabetes. This may relate to certain south asian populations (54)."

Reviewer #3: The authors attempted to resolve an important problem in the field: accessing the beta-cell carrying capacity.

We thank the reviewers for this endorsement.

My major concern about this manuscript is whether the proposed HOMA-C is a good index for carrying capacity. By the definition of carrying capacity, it should be a constant for each adult individual, while HOMA-C varies with glucose and insulin levels. Scrutinizing the formula of HOMA-C, I suspect HOMA-C resembles HOMA-B, and the conclusions derived with HOMA-C can be obtained directly with HOMA-B. To rule out this possibility, I suggest the authors replot Figure 1 by replacing HOMA-C with HOMA-B and compare with the original Figure. For example, the author states that HOMA-C varies by a factor of about 10 between individuals with a CV of about 75%. Then, how much does HOMA-B vary among individuals?

Response:

We thank the reviewer for this helpful comment, which helped us to add comparison to additional HOMA indicators. We have now compared HOMA-C to HOMA-B. We find that HOMA-C varies about twice as much between individuals than HOMA-B. We replot Fig 1 with HOMA-B in the new SI figure S1.

We also compared the ability of HOMA-C and HOMA-B to predict transition to diabetes after one year. We find that HOMA-C outperforms HOMA-B. The mean risk per quartile is 2.1 for HOMA-C and 1.3 for HOMA-B. This risk dependence is calculate through:

$$bootstrap\_mean\{mean\{\frac{risk_{q1}}{risk_{q2}},\frac{risk_{q2}}{risk_{q3}},\frac{risk_{q3}}{risk_{q4}}\}\}$$

We also evaluated the intrapersonal CV divided by the intrapersonal CV, a measure of how much HOMA-C and HOMA-B vary over time compared to over the population. We find that HOMA-B does vary widely within a person compared to the population

variance, with an intra/inter ratio of about 1:2.5, slightly more variable than HOMA-C with a 1:3 ratio.

We conclude that in our cohort, HOMA-C varies between individuals more than HOMA-B, is slightly more relatively stable over time, and that it is a better predictor of transition to diabetes than HOMA-B.

We now add these results to the text, summarizing the risk analysis for 6 measures (Fig. 2, line 786):

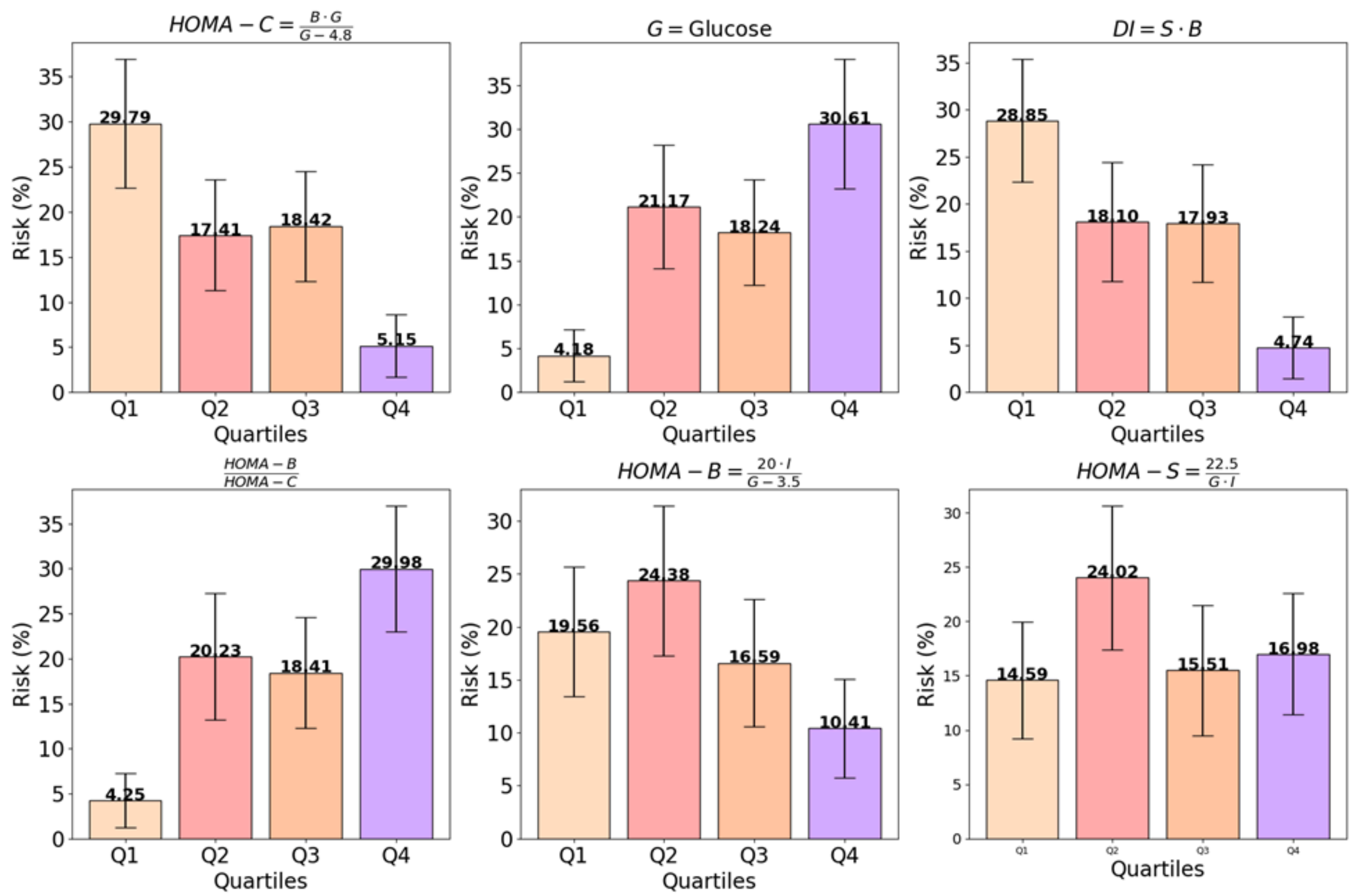

Figure 2. Correlation between diabetes transition risk and diabetes related measures – HOMA-C, G, HOMA-DI, HOMA-B/HOMA-C, HOMA-B, HOMA-S. The bars show, for each parameter, the frequency of transitioning to diabetes over a year in each quartile of the parameter. The mean risks and confidence intervals were calculated using bootstrapping. The bars are colored by quartile order. Beneath each graph are the Pearson correlation values, the risk ratio between the highest and lowest quartiles, and the average risk ratio between quartiles [q1/q2, q2/q3, q3/q4].

In the discussion we add (line 404): “The estimator HOMA-C is composed of fasting glucose and insulin tests which vary over time. In order to remain roughly constant in an

individual, these tests need to vary in a way that keeps HOMA-C constant. We find that the intra-personal CV relative to the interpersonal CV of HOMA-C is relatively small (~25%). Other measures such as HOMA-B, S and DI show a somewhat wider variation (Fig. S1) - ~38%, 52% and 71% respectfully.”

In lines 39 and 40, the authors state, “Unlike previous mathematical models, the new model can explain the slow rise in glucose over decades”. However, many other diabetes progression models can explain the slow rise in glucose over decades. For example, in the work of Yang et al [1], the proposed model can capture the trend of the longitudinal glucose data of the Pima Indian tribe, including the slow progression during the prediabetic stage.[NT27] \

Response: We have now corrected the text. Yang et al also use a compensation mechanism that limits beta cell function. This claim was removed, and we refer to papers which show models capturing the long term rise of T2D in line 384: “To explain prediabetes under the conditions where beta-cell growth is faster or similar to the rate at which insulin resistance rises, models need to have a limit to beta cell capacity. Several previous models used an insulin-based inhibition of beta cell function (17,49,50) or a pairwise interaction term with a free-fatty acid inflammation term (18). Without the constraint on the timescales, prediabetes rises in additional models (9,52), as demonstrated in Fig. 4.”

In lines 74 and 75, the authors state, “total ability of beta cells to secrete insulin is called the beta cell functional mass”. This is the new definition defined by the authors. It is called beta-cell function in the clinical setting.[NT28]

Response: We have now added this nomenclature (line 76): “The total ability of beta cells to secrete insulin is called the beta cell functional mass, also called beta-cell function in clinical settings”.

In lines 146 and 147, the authors state, “when B is much smaller than the carrying capacity… return to the Topp model”. The sentence should be rephrased because this model cannot return to the Topp model! One of the key features of the Topp model is that the model can switch from negative feedback to positive feedback when glucose surpasses a certain level. This feature requires two roots of the function on the right-hand side of the beta-cell mass equation.[NT29]

Response: We have now corrected this error by removing the comparison to Topp (line 152): "...there is sufficient compensation when *B* is much smaller than the carrying capacity."

In lines 451 and 452, the authors write,"…and somatostatin secreted by delta cells which reduces insulin release". Reducing insulin release doesn't imply changing the maximal secretion.[NT30]

Response: Correct insight, this part was removed.

In line 612, the authors need to show the reasoning steps for the equation G=δ⁄B^(1/3) .[NT31] [NT32]

Response: It was indeed missing. We removed the entire seasonality analysis from the paper, including these equations.

1. Yang, B., et al., Modeling the progression of Type 2 diabetes with underlying obesity. 2023. 19(2): p. e1010914.

Have the authors made all data and (if applicable) computational code underlying the findings in their manuscript fully available?

The PLOS Data policy requires authors to make all data and code underlying the findings described in their manuscript fully available without restriction, with rare exception (please refer to the Data Availability Statement in the manuscript PDF file). The data and code should be provided as part of the manuscript or its supporting information, or deposited to a public repository. For example, in addition to summary statistics, the data points behind means, medians and variance measures should be available. If there are restrictions on publicly sharing data or code —e.g. participant privacy or use of data from a third party—those must be specified.

Reviewer #1: Yes

Reviewer #2: Yes

Reviewer #3: Yes

PLOS authors have the option to publish the peer review history of their article (what does this mean?). If published, this will include your full peer review and any attached files.

If you choose "no", your identity will remain anonymous but your review may still be made public.

Do you want your identity to be public for this peer review? For information about this choice, including consent withdrawal, please see our Privacy Policy.

Reviewer #1: No

Reviewer #2: No

Reviewer #3: **Yes:** Boya Yang
