# Supplementary material for: Quantification of beta-cell carrying capacity in prediabetes
Source: PLoS Comput Biol. 2026 Sep 15;22(9):e1014589. doi: 10.1371/journal.pcbi.1014589 (PMC13638054; doi:10.1371/journal.pcbi.1014589)
Supplement: Supplementary file 2 [file pcbi.1014589.s009.pdf]

Dear Dr. Woller,

Thank you for submitting your manuscript to PLOS Computational Biology. The reviewers all appreciate the careful revision of this manuscript, where many of the concerns raised have been addressed. However, after careful consideration, we feel that this revision does not fully meet PLOS Computational Biology's publication criteria as it currently stands. Therefore, we invite you to submit a revised version of the manuscript that addresses the points raised during the review process.

Please submit your revised manuscript within 30 days Nov 16 2025 11:59PM. If you will need more time than this to complete your revisions, please reply to this message or contact the journal office at ploscompbiol@plos.org. When you're ready to submit your revision, log on to https://www.editorialmanager.com/pcompbiol/ and select the 'Submissions Needing Revision' folder to locate your manuscript file.

Please include the following items when submitting your revised manuscript:

* A rebuttal letter that responds to each point raised by the editor and reviewer(s). You should upload this letter as a separate file labeled 'Response to Reviewers'. This file does not need to include responses to formatting updates and technical items listed in the 'Journal Requirements' section below.
* A marked-up copy of your manuscript that highlights changes made to the original version. You should upload this as a separate file labeled 'Revised Manuscript with Track Changes'.
* An unmarked version of your revised paper without tracked changes. You should upload this as a separate file labeled 'Manuscript'.

If you would like to make changes to your financial disclosure, competing interests statement, or data availability statement, please make these updates within the submission form at the time of resubmission. Guidelines for resubmitting your figure files are available below the reviewer comments at the end of this letter.

We look forward to receiving your revised manuscript.

Kind regards,

Sunil Laxman, PhD
Academic Editor
PLOS Computational Biology

Mark Alber
Section Editor
PLOS Computational Biology

Response Letter - “Quantification of beta-cell carrying capacity in prediabetes”

We thank the reviewers and editor for their insights and corrections. The manuscript has been revised accordingly. Below we address your comments point-by-point, indicating the corresponding line numbers in the revised version.

Reviewer #1

Comment: The paper is much improved. Table 1 and Fig. 2 are valuable additions (Risk prediction with HOMAC). The new model is shown to be somewhat better at predicting diabetes than other models based on fasting measurements. Models based on responses to glucose challenges, such as OGTTs or MMTs, might be better, but the data used here do not permit testing this and fasting measures may be preferred for reasons of cost and patient burden anyway.

Response: We thank the reviewer for this positive assessment. We now acknowledge in the Discussion (L. 487–490): "Different predictive models based on dynamic responses to glucose challenges, such as OGTTs or mixed-meal tests, might ultimately provide better risk estimates; however, the data analyzed here did not include such tests, and fasting measures remain preferable for reasons of cost, simplicity, and patient burden."

Comment: The revised version does a much better job also of putting the new model in context with other current models. I still feel, however, that it overstates the big divide between carrying capacity and glucotoxicity (e.g., the text between L. 381 and 392 in the Discussion). Carrying capacity is an abstraction and may overlap at the cellular level with metabolic stress caused by high glucose load. Experimental data are not currently sufficient to know the relative importance of the two. All existing models, including this one, agree that T2D results from inadequate capacity to compensate for insulin resistance. This could happen either because insulin resistance develops rapidly or because the ability to increase beta-cell function is limited or any combination of those.

Response: We agree, and have revised the relevant passage (L. 383–387) to state that carrying capacity is not essential for diabetes in a mathematical model:
”Models without a carrying-capacity constraint can also reproduce prediabetes and diabetes when β-cell compensation is sufficiently slow compared to the rate of increase in insulin resistance (9, 52), as shown in Fig. S5. However, this rate-dependence also exists in models with a carrying capacity; the main difference being that with CC, prediabetes emerges even at very fast rates of compensation (relative to the rise rate of IR).”

Comment: As an example, people with low carrying capacity can develop prediabetes and diabetes even if insulin resistance is slow and modest in degree. This is the case for Koreans (PMID: 26577716) and likely for other East Asian populations, as noted by Reviewer #2.

Response: Added this reference and example in the Discussion (L. 471).

Major Comments

Comment: Fig. 3 is not described in Results, only mentioned in passing at L. 372 in the Discussion. Fig. 3 and Fig. 4 should be swapped, or perhaps Fig. 3 should be made a Supplementary Figure.

Response: We made it a supplementary figure, sensei t is barely referred to in the text.

Comment: L. 76, 117 "hypertrophy": Hyperplasia (more beta cells) probably plays a much bigger role than hypertrophy (bigger cells). Reference #22 about senescence is intriguing, but we don't know whether beta cells are more able to increase cell size than cell number in adulthood. The uncertainty should be acknowledged.

Response: We thank the reviewer for correcting this point. We revised both sections (L. 76–80, 125–126), accordingly:
L.77-80: “Increased beta cell mass in humans likely reflects hypertrophy and/or hyperplasia, depending on the physiological context. Current thought is that until adulthood mass change is mainly due to hypertrophy (21), and studies suggest that increases in β-cell mass with obesity and pregnancy involves increased cell number (55,56,57). “
L.125-126: “Human data suggest that total beta cell mass can double in obesity (23,24,13,25) and pregnancy (26), where studies suggest the mechanism to be an increased cell number (55,56,57).”

Comment: L. 289: Say in this paragraph that the phase plane analysis uses the assumption that I is much faster than G, as discussed in Materials and Methods. This assumption was not needed for the derivation of HOMA-C, but it is needed here.

Response: Added this clarification at L. 298–299: “The dynamics described in this phase plane analysis include the assumption that insulin -dynamics are much faster than Glucose dynamics, as discussed in Materials and Methods.”

Comment: L. 307 - 309, Fig. 4C: Why does the response to a step reduction in SI (a maintained increase in glucose) differ from the original Topp model (return to baseline)? Does this just depend on the size of the drop in SI or the value of the CC parameter C? How is the value of C chosen?

Response: This is indeed a key feature of the model with CC. Given some external drive to the system such as a drop in SI, compensation occurs to bring the system to its previous fixed point, but in this model compensation does not fully occur. Beta-cell functional mass cannot increase as required to maintain normoglycemia, due to the limitation of the CC. So, indeed, a change in a parameter results in partial compensation, whose magnitude depends on the new value of SI and the CC.

We add to the text:
L. 316-320: “For example, upon a hypothetic step-like rise in insulin resistance, the model shows how glucose rises within hours. As beta cell mass increases over weeks, fasting

glucose slowly declines, but there is only partial beta-cell compensation in the CC model, such that fasting glucose declines to a higher value than before, producing a prediabetic stable state (Fig. 3C)."

Fig. 3C: "Dynamics of the carrying capacity model for a step-like decrease in insulin sensitivity from to , an arbitrary magnitude to qualitatively reproduce an effect similar to the step-change the original Topp model. A key feature of the CC model, is that glucose steady state changes when changing SI – only partial compensation occurs, resulting a prediabetic state instead of a return to normoglycemia."

Comment: L. 335 - 336, Fig. 4F: "Notably, the Topp model without carrying capacity has different nullclines that cannot capture these subtypes of diabetes." Please modify to "... the linearized Topp model without glucotoxicity or carrying capacity ...".

Response: Changed accordingly at L. 344 and Fig. 3F. - "the linearized Topp model without glucotoxicity…".

Comment: L. 364, "We claim that it is unlikely that beta cell hypertrophy takes several years, and thus compensation rate is not much slower than the timescales for insulin resistance ": I prefer "assume" to claim, since this is not known for humans. This could vary greatly among individuals and populations. In a study of Zucker Diabetic Fatty Rats (PMID: 17895283) it was shown (for the females) that slow rise of insulin resistance was compensated, but fast rise led to diabetes. This contrary evidence should be cited.

Response: We agree. These lines were removed in order to make the discussion less repetitive around the saying that "prediabetes rises when beta-cell compensation fails to catch up with the rate of change of insulin resistance" which we felt appeared in different forms.

Comment: L. 366, "mathematical models of compensation without carrying capacity or any compensation limitation, cannot explain the slow rise of glucose in prediabetes." Please add "unless compensation is slower than insulin resistance" here even though this point is made later.

Response: We agree. These lines were removed in order to make the discussion less repetitive around the saying that "prediabetes rises when beta-cell compensation fails to catch up with the rate of change of insulin resistance" which we felt appeared in different forms.

Comment: L. 371, "Yet, if beta-cell compensation times are slow enough, prediabetes and diabetes are obtained in models without CC (such as 9, 52), as demonstrated in Fig. 3": This statement is misleading. Models with carrying capacity also show that the rate of increase of glucose and the risk of crossing the threshold to diabetes rise when the rate of rise of insulin resistance is greater. Conversely, diabetes risk should increase for any given rate of rise of insulin resistance if carrying capacity is lower.

Response: We agree. These lines were removed in order to make the discussion less repetitive around the saying that “prediabetes rises when beta-cell compensation fails to catch up with the rate of change of insulin resistance” which we felt appeared in different forms.

Minor Comments

Comment: L. 58, "glucose exceeds 7 mM": should be "fasting glucose" - Fixed.

Comment: L. 99, "unmedicated": Was there also no life-style intervention (diet and/or exercise)?

Response: Yes, there was. Added (L.105-106): “...under a dietary intervention.”

Comment: L. 59 - 60, "Since type-2 diabetes is associated with morbidity and mortality": Prediabetes is also associated with morbidity and mortality. The main reason for treating prediabetes is that it is much easier to reverse than diabetes.

Response: Very true, changed L.59-60 to: “Since type-2 diabetes and prediabetes are associated with…”

Comment: L. 63, "removing glucose from the blood": add "and suppressing release from the liver". - Added.

Comment: L. 63, "each unit of insulin": Delete "each unit of". -Done.

Comment: L. 277, Table 1: Say here or at line 272 of the text that the q_i are the quartiles. The phrase "mean risk factor per ratio" was unclear to me.

Response: We have now clarified the text and added at L.285 (equivalent of L.277 in the previous version): “...where q_i are the quartiles”.

Comment: L. 300, "Some I conditions": Change to "Some insulin conditions" as stated in the rebuttal. -Done.

Comment: L. 336: Say "the simplified Topp model" rather than "the Topp model" - Done.

Comment: Footnote after L. 423: "HOMA-B is proportional to 1/I" should be "HOMA-S is proportional to 1/I". - A necessary correction. Done.

Comment: L. 462: I think Ref #26 should be #29. - Done.

Comment: L. 484, "Insulin is secreted by beta cells with mass beta cell mass ": should be "Insulin is secreted by beta cells with functional mass ". - Done.

Comment: L. 592, 597: Which equation, #16 or #17, was used to make Fig. 4H?

Response: This was unclear. We modified the text of Fig. 3H (previously Fig. 4H): "The dynamics of the glucotoxicity model (eq. 16) for a slow exponential increase in insulin resistance".

Comment: L. 845, legend for Fig. 4, panel F: Say "the simplified Topp model" rather than "the Topp model" - Done.

Reviewer #2
Comment: None.

Reviewer #3
Comment: I appreciate the authors' efforts in comparing HOMA-C and HOMA-B. However, it is not demonstrated whether the conclusion drawn from HOMA-C in Figure 1 could (or not) be obtained using HOMA-B. While Figure 2 highlights that HOMA-C is a stronger predictor of diabetes transition risk, the comparison of correlations with transition risk alone does not convincingly establish that HOMA-C reflects the concept of "maximal carrying capacity." In addition, the bootstrapping method used in Figure 2 is not clearly explained and may confuse readers.

Most importantly, the statement "In order to remain roughly constant in an individual, these tests need to vary in a way that keeps HOMA-C constant" suggests that the authors expect HOMA-C to remain relatively stable within individuals, consistent with its proposed definition. However, given the formula for HOMA-C, it is not clear whether this stability can be achieved in practice. I recommend that the authors present individual trajectories of HOMA-C during disease progression. Demonstrating that HOMA-C shows only slight variation within individuals from the prediabetic stage onward would strengthen the argument and provide reassurance to readers regarding its biological interpretation.

Response: This is an important point and we thank the reviewer for noticing it. The response to that is that HOMA-C proves not to be a constant of an individual on one hand, and on the other hand it is relatively stable within an individual compared to the inter-individual variance. This is similar for HOMA-C as well, the main difference between the two being HOMA-C's predictive power for the risk of transitioning to diabetes in this cohort which exceeds that of HOMA-B.
We added to the discussion:
"HOMA -C is more stable within a person (Fig. S1B) than glucose or HOMA-DI in our cohort. HOMA-B is similarly constant over time but is less predictive of diabetes. Close to the diabetes threshold, DI and G may be better predictors than HOMA-C. Further work can test whether HOMA -C might be a better predictor for prediabetic patients on longer timescales of 5 or 10 years."

We follow the suggestion and plot the longitudinal trajectories of each parameter in Fig.S4, showing a better relative stability in HOMA-C and HOMA-B compared to G and DI, where HOMA-S's stability resides between these two groups:

"

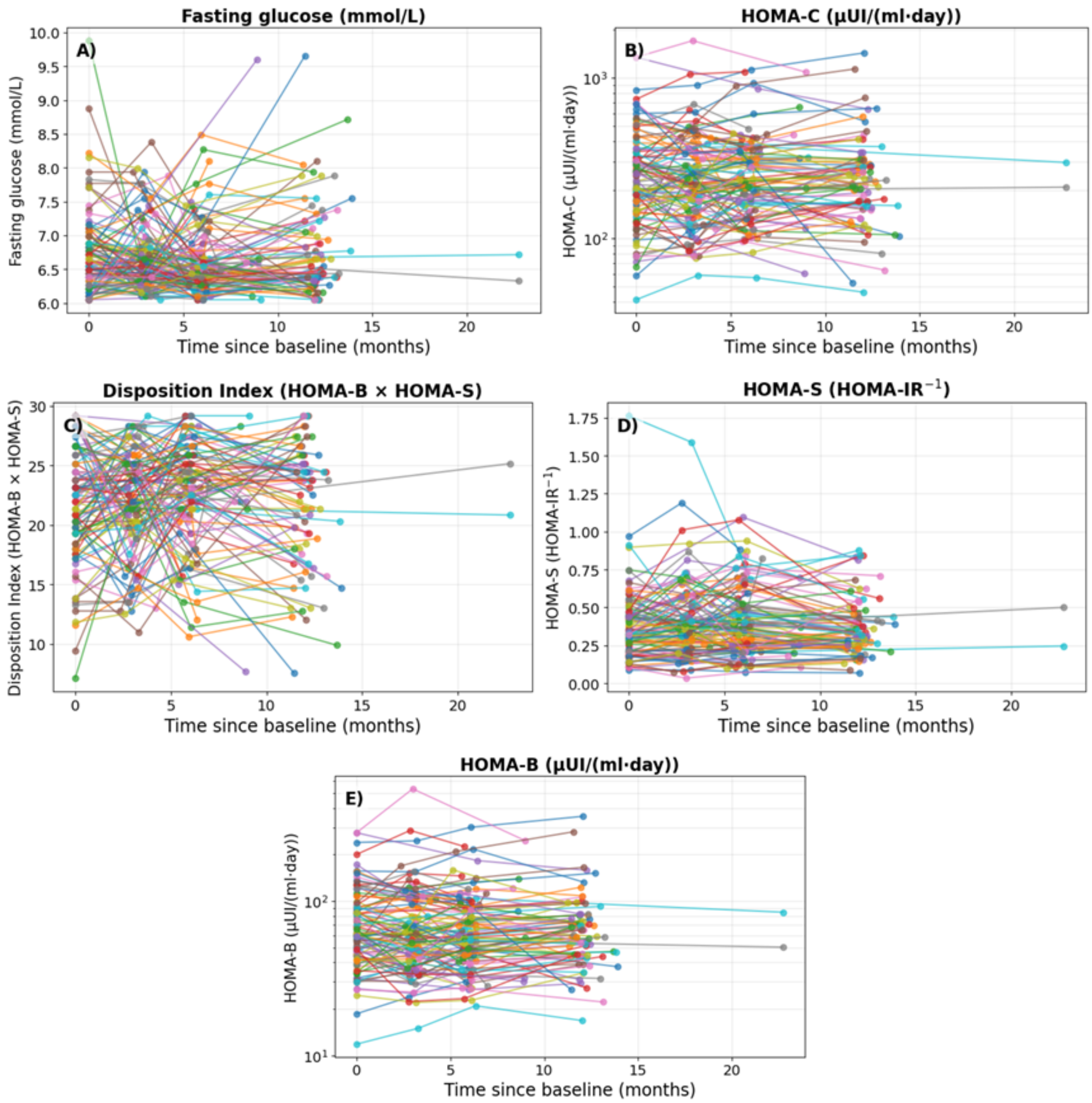

Figure S4. Longitudinal trajectories of the cohort throughout the measurement period, for different HOMA- parameters. 109 trajectories were taken, with an initial fasting glucose greater than 6 mmol/L to focus on the change of the parameters during prediabetes and diabetes. For each parameter, the mean intra- and inter-individual CVs, and their ratio, are computed. HOMA-B,C,S are more constant along the year of measurement than DI,G, and HOMA-B,C have a lower intra- to inter-personal variance ratios.

(A) Fasting glucose. Intra CV 4.6%, inter CV 6.2%, ratio: 0.74.

(B) HOMA-C. Intra CV 21.3%, inter CV 71%, ratio: 0.3.

(C) Disposition index. Intra CV 13.5%, inter CV 15.3%, ratio: 0.88.

(D) HOMA-S. Intra CV 19.7%, inter CV 50.3%, ratio: 0.39.

(E) HOMA-B. Intra CV 17.5%, inter CV 66%, ratio: 0.27. "

We added in the main text, L.247-251:

"Other measures such as HOMA-B, S and DI show an inter-to-intrapersonal variation ratio of ~35%, 50% and 70% respectfully (Fig. S1B), which are somewhat wider relative to HOMA-C, except for HOMA-B which varies in a similar magnitude. We show individual trajectories of HOMA-C, glucose, HOMA-B, HOMA-DI and HOMA-S in Fig S4, where the CVs are presented again after screening for high fasting glucose."

In addition, we have made the quartile risk calculation more clear. We now provide a detailed description in the Materials and Methods section (L. 574–586):

"For each participant, we calculated the parameters HOMA-B = 20 I / (G − 3.5), HOMA-S = 22.5 / (G I), HOMA-C = G HOMA-B / (G − 4.8), the disposition index DI = HOMA-B × HOMA-S, and the ratio HOMA-B / HOMA-C, using the first available fasting test.

To estimate uncertainty in the risk curves shown in Figure 2, the dataset was resampled with replacement 1 000 times. In each resample, participants were divided into quartiles according to each parameter. Within each quartile, we computed the fraction of participants who transitioned to diabetes, representing the empirical transition risk. The mean and standard deviation of this risk across bootstrap iterations were used to plot the bars and error ranges in Figure 2.

For a compact numerical comparison (Table 1), we defined a mean risk-ratio index as the average ratio of successive quartile risks, Q_i:

$$(18)\quad mean\{\frac{Q_1}{Q_2}, \frac{Q_2}{Q_3}, \frac{Q_{31}}{Q_4}\}$$

This value was sampled over bootstrapping iterations and its mean and standard deviation are shown in Table 1.

This index summarizes how sharply transition risk decreases across quartiles of each parameter, or increases, if the parameter positively affects transition risk."
