# Supplementary material for: Quantification of beta-cell carrying capacity in prediabetes
Source: PLoS Comput Biol. 2026 Sep 15;22(9):e1014589. doi: 10.1371/journal.pcbi.1014589 (PMC13638054; doi:10.1371/journal.pcbi.1014589)
Supplement: Supplementary file 3 [file pcbi.1014589.s010.docx]

**Additional Editor Comments:**

There remain some key points of concern. One is related to the text itself - where some changes (indicated in the response letter) do not manifest in the manuscript text, and also includes one missing reference, and one incorrect statement related to a reference. It is essential for this to be corrected.

The second is related to valid concerns raised by one reviewer, regarding bench marking or comparing against HOMA-B. Given that HOMA-B is widely used for b-cell insulin producing capacity, the reviewer raises valid concerns on the need for some comparison of HOMA-C with HOMA-B, to allow readers to assess whether this index provides a useful, alternate representation of b-cell carrying capacity. It will be critical to provide some feedback on this point - potentially in the results section, but more extensively in the discussion.


**Reviewers' comments:**

Reviewer's Responses to Questions

**Comments to the Authors:
Please note here if the review is uploaded as an attachment.**

Reviewer #1: Almost all the requests raised in the second review have now been answered. However, two of the claimed changes do not actually appear in the MS, as listed below, along with one other minor correction.

Response: We thank the reviewer for noting these mistakes in the text, and follow to correct them in the MS:

1. “Models without a carrying-capacity constraint ... (9. 52), as shown in Fig. S5”

These sentences are not in the MS. Similar words are found at l. 425 - 426 but call out Fig. S6, not S5, which seems correct:

“Without the constraint on the timescales of IR rise, prediabetes arises in additional models (9,52) as shown in Fig. S6.”

Response: The correct lines are 435-437, with a shorter and more concise phrasing, and the figure number been changed to S5 which is the correct figure:

“Without the constraint on the timescales of IR rise, prediabetes arises in additional models (9,52) as shown in Fig. S6. We note that a slow rise in IR e is not essential in the present model for prediabetes. “

2. "Added this reference and example in the Discussion (L. 471)".

The reference (Ohn et al, PMID: 26577716) was not added.

Response: We add the missing referrence in L. 411-413:

“In addition, ref. (58) points that for Koreans in their study, T2D and prediabetes can develop even if insulin resistance is slow and modest in degree, for people with low carrying capacity.”

3. Hypertrophy vs. hyperplasia. L. 77 - 80: "Current thought is that until adulthood mass change is mainly due to hypertrophy (21)":

Ref. 21 says the opposite, that hyperplasia is dominant until adulthood.

Response: We thank the reviewer for pointing out this error, which has now been fixed – hypertrophy changed to “hyperplasia” according to the paper.

Reviewer #3: The authors use HOMA-C to define “beta-cell carrying capacity,” but report that HOMA-C has higher intra- and inter-CV compared with HOMA-B (see the caption of Figure S4). This raises doubts about whether readers will accept an unstable index as a valid representation of beta-cell carrying capacity.
Furthermore, the authors have not demonstrated whether the conclusions drawn from HOMA-C in Figure 1 could also, or could not, be obtained using HOMA-B. This avoidance reinforces my concern about whether the proposed index HOMA-C meaningfully contributes to the field.

Response: The reviewer notes that HOMA-B is somewhat more stable than HOMA-C. We agree. The reviewer than says that HOMA-C is unstable. We do not agree- , both HOMA-B and HOMA-C are very stable (CV intra=0.25 which is a third of CV inter=0.75) compared to other indices like insulin resistance and disposition index (CV intra=0.13 which is close to CV inter=0.15). Furthermore HOMA-C is a better predictor than HOMA-B for diabetes onset. Therefore, we conclude that HOMA-C is a stable index that has predictive value.

We now note this in the discussion, L. 386-390:

“Both HOMA-C and HOMA-B are stable in the sense that their intrapersonal CV is about a third of their interpersonal CV. This is in contrast to other indices such as insulin resistance and disposition index where the interpersonal and intrapersonal CV are 0.4 and 0.9 respectively. Since HOMA-C is a better predictor of diabetes than HOMA-C, we conclude that it is a relatively stable index useful for prediction.“
