# Supplementary material for: Quantification of beta-cell carrying capacity in prediabetes
Source: PLoS Comput Biol. 2026 Sep 15;22(9):e1014589. doi: 10.1371/journal.pcbi.1014589 (PMC13638054; doi:10.1371/journal.pcbi.1014589)
Supplement: Supplementary file 4 [file pcbi.1014589.s011.docx]

Dear Editor,

We have now addressed all of reviewer 3 comments. As presented below.

The main changes added were clarifications and temperament of specific points.

**Comment**: While the authors argue that HOMA-C is “stable” based on the ratio of intra- to inter-individual variability, I remain unconvinced that this supports its interpretation as a meaningful physiological construct. The reported coefficient of variation (CV intra ≈ 0.25 vs CV inter ≈ 0.75) does not by itself establish biological stability, as similar behavior can arise from mathematically constrained or model-derived quantities.

***Response****: We agree and have added a tempering statements in the Results, L. 252-253, and in the Discussion, L. 390-399.*

*L. 252-253: “… although a low intrapersonal-to-interpersonal CV ratio does not by itself establish that carrying capacity is a fixed physiological trait”.*

*L. 390,391: “is more stable” and “are stable” changed to “appears to be…”.*

L. 392: added “*although low interpersonal versus intrapersonal CV is not by itself proof of temporal stability”.*

L. 395-397: Deleted “*Since HOMA-C is a better predictor of diabetes than HOMA-C, we conclude that it is a relatively stable index useful for prediction. Close to the diabetes threshold, DI and G may be better predictors than HOMA-C.”*

*This leaves only the next line, which is more tempered: ”Further work can test whether HOMA -C might be a better predictor for prediabetic patients on longer timescales of 5 or 10 years.”*

**Comment**: More importantly, the central concern raised previously remains insufficiently addressed: it is still unclear whether HOMA-C provides information beyond that already contained in HOMA-B. Based on the revised figures (including Fig. S1), the qualitative conclusions drawn from HOMA-C in Figure 1, appear reproducible using HOMA-B alone. If so, HOMA-C may largely represent a reparameterization of the same fasting glucose–insulin relationship rather than a distinct or independently informative metric. Finally, the claim that HOMA-C reflects an intrinsic “carrying capacity” of beta-cell function remains conceptually problematic, as the quantity is derived from state-dependent variables (fasting glucose and insulin) and may therefore reflect the current metabolic environment rather than a fixed physiological limit. I encourage the authors to temper the interpretation of HOMA-C accordingly.

***Response****: We agree that this point can be better clarified. We now add to the manuscript in the discussion, L. 400-409:*

*"One crucial difference between HOMA-B and HOMA-C is that HOMA-C is undefined at euglycemic conditions, G<4.8mM, whereas HOMA-B is well defined.  HOMA-C depends on an out-of-homeostasis condition, namely prediabetes, to ascertain maximal beta cell capacity. It is built on the idea of a gap between insulin produced and insulin that would have been enough to return to homeostasis – prediabetes acts as a kind of stress to test the system and reveal its carrying capacity , which is hidden when the system is able to compensate and reach homeostasis. In this way, although it is based on state-dependent variables and not on a dynamic stress test like OGTT, we believe that HOMA-C carries information about carrying (maximal) capacity, in contrast to the current capacity measured by HOMA-B.  Our main reason for believing this is the mathematical model presented in the paper."*

*When discussing Fig1, we also add in L. 224-225:*

*"Note that HOMA-B and HOMA-C are not equivalent – for example HOMA-B of 100 corresponds to individuals with HOMA-C ranging from about 500 to 1500."*

*We add the following caveat in L. 298-300:*

*“…though, as noted in the Discussion, this predictive association does not by itself distinguish whether HOMA-C captures information beyond a reparameterization of fasting glucose and insulin.”*

*We also temper our calims throughout. In the discussion, L. 456-457, we add:*

*"HOMA-C differs from HOMA-B in applying only to the prediabetes state, and its interpretation is based on a mathematical model and hence requires experimental testing."*

*In the abstarct we add in L. 41-42:*

*"The interpretation of HOMA-C as a carrying capacity relies on a mathematical model and requires further experimental testing."*

*And change in L.42:*

*“The present quantification of beta cell carrying capacity…” to “Quantification of beta cell…”*

*We further differentiate the concept of HOMA-C in the abstract, L. 35-37:*

*“It uses prediabetes as a stress and estimates carrying capacity using the gap between secreted insulin and the amount of insulin needed for homeostasis.”*

*In addition, in the author summary, L. 56-57 we add:*

*“We note that further experimental testing is required to understand HOMA-C and its distinction from HOMA-B.”*
