# Supplementary material for: Quantification of beta-cell carrying capacity in prediabetes
Source: PLoS Comput Biol. 2026 Sep 15;22(9):e1014589. doi: 10.1371/journal.pcbi.1014589 (PMC13638054; doi:10.1371/journal.pcbi.1014589)
Supplement: Peer review history

# Peer review: Decision Letter 0

**DOI:** 10.1371/journal.pcbi.1014589.r001

**Authors:** Jason Haugh, Sunil Laxman

17 Jul 2024

Dear Dr Woller,

Thank you very much for submitting your manuscript "Quantification of beta cell carrying capacity in prediabetes" for consideration at PLOS Computational Biology.

As with all papers reviewed by the journal, your manuscript was reviewed by members of the editorial board and by several independent reviewers. In light of the reviews (below this email), we would like to invite the resubmission of a significantly-revised version that takes into account the reviewers' comments. Additionally, there are several concerns about possibly over-extrapolating the well known model by Topp, as well as what seems like some strawman arguments that have been made. These should be carefully addressed.

We cannot make any decision about publication until we have seen the revised manuscript and your response to the reviewers' comments. Your revised manuscript is also likely to be sent to reviewers for further evaluation.

When you are ready to resubmit, please upload the following:

[1] A letter containing a detailed list of your responses to the review comments and a description of the changes you have made in the manuscript. Please note while forming your response, if your article is accepted, you may have the opportunity to make the peer review history publicly available. The record will include editor decision letters (with reviews) and your responses to reviewer comments. If eligible, we will contact you to opt in or out.

[2] Two versions of the revised manuscript: one with either highlights or tracked changes denoting where the text has been changed; the other a clean version (uploaded as the manuscript file).

Important additional instructions are given below your reviewer comments.

Please prepare and submit your revised manuscript within 60 days. If you anticipate any delay, please let us know the expected resubmission date by replying to this email. Please note that revised manuscripts received after the 60-day due date may require evaluation and peer review similar to newly submitted manuscripts.

Thank you again for your submission. We hope that our editorial process has been constructive so far, and we welcome your feedback at any time. Please don't hesitate to contact us if you have any questions or comments.

Sincerely,

Sunil Laxman, PhD

Academic Editor

PLOS Computational Biology

Jason Haugh

Section Editor

PLOS Computational Biology

***********************

Reviewer's Responses to Questions

**Comments to the Authors:**

**Please note here if the review is uploaded as an attachment.**

Reviewer #1: This paper explores the impact of assuming a limitation in carrying capacity (CC) in beta-cell functional mass. A modified version of the Topp model is used (ref. 9). CC has been incorporated in other models of glucose-insulin homeostasis and diabetes derived from Topp (e.g, cited refs. 17, 18), but as a secondary effect to the main player, glucotoxicity. The present paper shows that CC in the absence of glucotoxicity can lead to prediabetes and diabetes and that a crippled version of Topp without glucose toxicity or CC cannot lead to disease (Fig. 4A). In other words, CC is shown to be sufficient but is not shown to be necessary for disease.

The new model also assumes that beta-cell compensation to insulin resistance is much faster than insulin resistance itself, which permits reduction of the three-variable system to two dimensions and application of phase-plane analysis. I find the assumption questionable, though they show data that is supportive and some of the conclusions that follow dubious. The authors should at least acknowledge that this assumption is radically different from other models of this class.

Specific Comments:

L. 99 - 100, “carrying capacity … does not vary strongly with age”: Most of the data shown for this is cross-sectional, and the small set of longitudinal data only covers one year. It seems unlikely that a quantity based on fasting insulin and glucose would not change markedly over longer time periods. This statement needs to be qualified.

L 127 Eq. 3: the linearized Topp model predicts exponential growth without limit. Linearization is justified by saying that the increases in glucose during pre diabetes are well below the threshold where positive feedback leading to diabetes is set in Topp (L. 510). The Topp model is poorly calibrated, but other models have shown that glucotoxicity is by itself sufficient for prediabetes.

L. 235 - 245: Carrying capacity is shown to predict transition from prediabetes to diabetes. However, the curves of constant CC in Fig. 1B are very similar to curves of constant disposition index defined by HOMA IS * HOMA B. Disposition indices based on various measures of insulin sensitivity and beta-cell function are good detectors and predictors of diabetes, and I believe that the favorable properties shown for HOMA C may arise from its similarity to a disposition index. To be convinced otherwise, I would need to see evidence that HOMA C is a better predictor than the HOMA disposition index or HOMA B, which it also closely resembles.

L. 280 - 294, postpartum recovery of glucose: This is one of the datas sets used to justify that beta-cell compensation is much faster than insulin resistance. The change in glucose is only 0.14 mM, about 3%. I question whether such small changes shed meaningful light on the much bigger changes as people progress to prediabetes.

L. 323 - 4 and Fig. 4A, “the classic Topp model … can’t explain prediabetes: What is shown is that the linearized Topp model is unable to explain prediabetes. This is trivially true, since functional mass can grow without bound. Even in the Topp model with glucotoxicity (used in Fig. 5G), the predicted increase in mass is implausibly large, but other better calibrated models with glucotoxicity but without CC can account for prediabetes, for example Fig. 7 in Ref. 9 and Fig. 2 in https://pubmed.ncbi.nlm.nih.gov/32663101. The same applies to L 431 - 432 in the Discussion.

L. 369 and Fig. 5C: the response to a step-wise reduction in Si is shown to result in prediabetes. However, prediabetes results from a slow decline in Si over time, not a step. Topp-like models predict that after a step, glucose will come back to baseline. That seems like the right prediction, but I don’t know of any data to settle the question. More important, as stated above, when Si declines slowly, the Topp model and its successors can exhibit prediabetes, so the example of Fig. 5C does not refute Topp-like models or demonstrate the necessity of CC.

L 414 and Fig. 5G: While the reduced Topp model with carrying capacity and insulin set to steady state shows a saddle-node bifurcation, in the original Topp model, with glucotoxicity but no CC, the transition to diabetes is not through an SN. See https://pubmed.ncbi.nlm.nih.gov/26300065/. The transition is more complicated. This casts doubt on the validity of the phase plane analysis.

L. 439 - 440: “Insulin sensitivity decreased by a factor of about 2 over 36 months”: This is much slower than the time scale in Figure 2. Please comment.

L. 512: Setting insulin to steady state in the phase plane analysis of Fig. 5 is justified by the clearance rates of insulin and glucose. But if insulin is really faster than glucose, why does it lag behind glucose in glucose tolerance tests?

Minor Points:

L. 46, “a low number of insulin-producing beta cells, called cell carrying capacity”: this is not correct. CC is a limitation in the ability to expand cell number or functional mass, now low number per se.

L. 360, “Some I changes”: seems like a typo.

L.378, “Fig. 5C”: should be 5D.

L. 387, ref. 37: should be 39.

L. 393, ref. 38: should be 40.

L. 450, “sulforea” is misspelled.

L. 462, “reduces by about 1.7 per quartile of HOMA-C”: I don’t understand what this number means. Also, cite the study it is based on.

Reviewer #2: In a rather clever move the authors extend the Topp model to add a

carrying capacity term in the B equation. This allows them to explain

prediabetes, which as the authors rightly point out, was not explained

in the original Topp model. The authors show this is a useful

extension by explaining NHANES data and diabetes subtypes as

well. This is a welcome addition to the HOMA literature as well,

because the authors are able to introduce a new index HOMA-C to deal

with prediabetes.

I must say that I was a somewhat surprised by the timescales that the

authors show for changes in beta-cell mass as well as insulin

resistance. I also note that the HOMA model used in the paper is the

classical, older version; nowadays the iHOMA model(s) is often claimed

to be "better".

I would like to ask the authors the following question. There are

populations such asin southeast Asia where it is claimed that (i)

about 50% of people that become prediabetic will go on to develop overt

diabetes, and (ii) typically within three years, say. How would you

explain these observations within your framework?

Reviewer #3: The authors attempted to resolve an important problem in the field: accessing the beta-cell carrying capacity. My major concern about this manuscript is whether the proposed HOMA-C is a good index for carrying capacity. By the definition of carrying capacity, it should be a constant for each adult individual, while HOMA-C varies with glucose and insulin levels. Scrutinizing the formula of HOMA-C, I suspect HOMA-C resembles HOMA-B, and the conclusions derived with HOMA-C can be obtained directly with HOMA-B. To rule out this possibility, I suggest the authors replot Figure 1 by replacing HOMA-C with HOMA-B and compare with the original Figure. For example, the author states that HOMA-C varies by a factor of about 10 between individuals with a CV of about 75%. Then, how much does HOMA-B vary among individuals?

In lines 39 and 40, the authors state, “Unlike previous mathematical models, the new model can explain the slow rise in glucose over decades”. However, many other diabetes progression models can explain the slow rise in glucose over decades. For example, in the work of Yang et al [1], the proposed model can capture the trend of the longitudinal glucose data of the Pima Indian tribe, including the slow progression during the prediabetic stage.

In lines 74 and 75, the authors state, “total ability of beta cells to secrete insulin is called the beta cell functional mass”. This is the new definition defined by the authors. It is called beta-cell function in the clinical setting.

In lines 146 and 147, the authors state, “when B is much smaller than the carrying capacity… return to the Topp model”. The sentence should be rephrased because this model cannot return to the Topp model! One of the key features of the Topp model is that the model can switch from negative feedback to positive feedback when glucose surpasses a certain level. This feature requires two roots of the function on the right-hand side of the beta-cell mass equation.

In lines 451 and 452, the authors write,“…and somatostatin secreted by delta cells which reduces insulin release”. Reducing insulin release doesn’t imply changing the maximal secretion.

In line 612, the authors need to show the reasoning steps for the equation G=δ B^(1/3) .

1. Yang, B., et al., Modeling the progression of Type 2 diabetes with underlying obesity. 2023. 19(2): p. e1010914.

**********

**Have the authors made all data and (if applicable) computational code underlying the findings in their manuscript fully available?**

The PLOS Data policy requires authors to make all data and code underlying the findings described in their manuscript fully available without restriction, with rare exception (please refer to the Data Availability Statement in the manuscript PDF file). The data and code should be provided as part of the manuscript or its supporting information, or deposited to a public repository. For example, in addition to summary statistics, the data points behind means, medians and variance measures should be available. If there are restrictions on publicly sharing data or code —e.g. participant privacy or use of data from a third party—those must be specified.

Reviewer #1: Yes

Reviewer #2: Yes

Reviewer #3: Yes

**********

PLOS authors have the option to publish the peer review history of their article (what does this mean?). If published, this will include your full peer review and any attached files.

If you choose “no”, your identity will remain anonymous but your review may still be made public.

**Do you want your identity to be public for this peer review?**  For information about this choice, including consent withdrawal, please see our Privacy Policy.

Reviewer #1: No

Reviewer #2: No

Reviewer #3: **Yes:** Boya Yang

Figure Files:

While revising your submission, please upload your figure files to the Preflight Analysis and Conversion Engine (PACE) digital diagnostic tool, https://pacev2.apexcovantage.com. PACE helps ensure that figures meet PLOS requirements. To use PACE, you must first register as a user. Then, login and navigate to the UPLOAD tab, where you will find detailed instructions on how to use the tool. If you encounter any issues or have any questions when using PACE, please email us at figures@plos.org.

Data Requirements:

Please note that, as a condition of publication, PLOS' data policy requires that you make available all data used to draw the conclusions outlined in your manuscript. Data must be deposited in an appropriate repository, included within the body of the manuscript, or uploaded as supporting information. This includes all numerical values that were used to generate graphs, histograms etc.. For an example in PLOS Biology see here: http://www.plosbiology.org/article/info%3Adoi%2F10.1371%2Fjournal.pbio.1001908#s5.

Reproducibility:

To enhance the reproducibility of your results, we recommend that you deposit your laboratory protocols in protocols.io, where a protocol can be assigned its own identifier (DOI) such that it can be cited independently in the future. Additionally, PLOS ONE offers an option to publish peer-reviewed clinical study protocols. Read more information on sharing protocols at https://plos.org/protocols?utm_medium=editorial-email&utm_source=authorletters&utm_campaign=protocols

# Author comment: Author response to Decision Letter 1

**DOI:** 10.1371/journal.pcbi.1014589.r002

13 Aug 2025

Attachment Submitted filename: Woller_et_al_Reviewers_Response.pdf

# Peer review: Decision Letter 1

**DOI:** 10.1371/journal.pcbi.1014589.r003

**Authors:** Mark Alber, Sunil Laxman

16 Sep 2025

PCOMPBIOL-D-24-00920R1

Quantification of beta cell carrying capacity in prediabetes

PLOS Computational Biology

Dear Dr. Woller,

Thank you for submitting your manuscript to PLOS Computational Biology. The reviewers all appreciate the careful revision of this manuscript, where many of the concerns raised have been addressed. However, after careful consideration, we feel that this revision does not fully meet PLOS Computational Biology's publication criteria as it currently stands. Therefore, we invite you to submit a revised version of the manuscript that addresses the points raised during the review process.

Please submit your revised manuscript within 30 days Nov 16 2025 11:59PM. If you will need more time than this to complete your revisions, please reply to this message or contact the journal office at ploscompbiol@plos.org. When you're ready to submit your revision, log on to https://www.editorialmanager.com/pcompbiol/ and select the 'Submissions Needing Revision' folder to locate your manuscript file.

Please include the following items when submitting your revised manuscript:

* A rebuttal letter that responds to each point raised by the editor and reviewer(s). You should upload this letter as a separate file labeled 'Response to Reviewers'. This file does not need to include responses to formatting updates and technical items listed in the 'Journal Requirements' section below.

* A marked-up copy of your manuscript that highlights changes made to the original version. You should upload this as a separate file labeled 'Revised Manuscript with Track Changes'.

* An unmarked version of your revised paper without tracked changes. You should upload this as a separate file labeled 'Manuscript'.

If you would like to make changes to your financial disclosure, competing interests statement, or data availability statement, please make these updates within the submission form at the time of resubmission. Guidelines for resubmitting your figure files are available below the reviewer comments at the end of this letter.

We look forward to receiving your revised manuscript.

Kind regards,

Sunil Laxman, PhD

Academic Editor

PLOS Computational Biology

Mark Alber

Section Editor

PLOS Computational Biology

**Additional Editor Comments :**

Please particularly address points raised by reviewer 3, regarding the presentation of individual trajectories of HOMA-C during disease progression, and emphasizing variations starting at the prediabetic stage. Additionally, some sections of the manuscript should present a more balanced viewpoint.

**Journal Requirements:**

1) We have noticed that you have uploaded Supporting Information files, but you have not included a complete list of legends. Please add a full list of legends for your Supporting Information files after the references list.

2) Please update your Data Availability Statement in the online submission form to include the link to the code.

3) Please amend your detailed Financial Disclosure statement. This is published with the article. It must therefore be completed in full sentences and contain the exact wording you wish to be published.

1) State what role the funders took in the study. If the funders had no role in your study, please state: "The funders had no role in study design, data collection and analysis, decision to publish, or preparation of the manuscript.".

Note: If the reviewer comments include a recommendation to cite specific previously published works, please review and evaluate these publications to determine whether they are relevant and should be cited. There is no requirement to cite these works unless the editor has indicated otherwise.

**Reviewers' comments:**

Reviewer's Responses to Questions

Reviewer #1: Overview

The paper is much improved. Table 1 and Fig. 2 are valuable additions (Risk prediction with HOMAC). The new model is shown to be somewhat better at predicting diabetes than other models based on fasting measurements. Models based on responses to glucose challenges, such as OGTTs or MMTs, might be better, but the data used here do not permit testing this and fasting measures may be preferred for reasons of cost and patient burden anyway.

The revised version does a much better job also of putting the new model in context with other current models. I still feel, however, that it overstates the big divide between carrying capacity and glucotoxicity (e.g., the text between L. 381 and 392 in the Discussion). Carrying capacity is an abstraction and may overlap at the cellular level with metabolic stress caused by high glucose load. Experimental data are not currently sufficient to know the relative importance of the two. All existing models, including this one, agree that T2D results from inadequate capacity to compensate for insulin resistance. This could happen either because insulin resistance develops rapidly or because the ability to increase beta-cell function is limited or any combination of those.

As an example, people with low carrying capacity can develop prediabetes and diabetes even if insulin resistance is slow and modest in degree. This is the case for Koreans (PMID: 26577716) and likely for other East Asian populations, as noted by Reviewer #2.

I have made some suggestions to make the view more balanced. I have made a lot of other suggestions, but these are mostly minor.

Major Comments

Fig. 3 is not described in Results, only mentioned in passing at L. 372 in the Discussion. Fig. 3 and Fig. 4 should be swapped, or perhaps Fig. 3 should be made a Supplementary Figure.

L. 76, 117 "hypertrophy": Hyperplasia (more beta cells) probably plays a much bigger role than hypertrophy (bigger cells). Reference #22 about senescence is intriguing, but we don't know whether beta cells are more able to increase cell size than cell number in adulthood. The uncertainty should be acknowledged.

L. 289: Say in this paragraph that the phase plane analysis uses the assumption that I is much faster than G, as discussed in Materials and Methods. This assumption was not needed for the derivation of HOMA-C, but it is needed here.

L. 307 - 309, Fig. 4C: Why does the response to a step reduction in SI (a maintained increase in glucose) differ from the original Topp model (return to baseline)? Does this just depend on the size of the drop in SI or the value of the CC parameter C? How is the value of C chosen?

L. 335 - 336, Fig. 4F: "Notably, the Topp model without carrying capacity has different nullclines that cannot capture these subtypes of diabetes." Please modify to "... the linearized Topp model without glucotoxicity or carrying capacity ...".

L. 364, "We claim that it is unlikely that beta cell hypertrophy takes several years, and thus compensation rate is not much slower than the timescales for insulin resistance ": I prefer "assume" to claim, since this is not known for humans. This could vary greatly among individuals and populations. In a study of Zucker Diabetic Fatty Rats (PMID: 17895283) it was shown (for the females) that slow rise of insulin resistance was compensated, but fast rise led to diabetes. This contrary evidence should be cited.

L. 366, "mathematical models of compensation without carrying capacity or any compensation limitation, cannot explain the slow rise of glucose in prediabetes." Please add "unless compensation is slower than insulin resistance" here even though this point is made later.

L. 371, "Yet, if beta-cell compensation times are slow enough, prediabetes and diabetes are obtained in models without CC (such as 9, 52), as demonstrated in Fig. 3": This statement is misleading. Models with carrying capacity also show that the rate of increase of glucose and the risk of crossing the threshold to diabetes rise when the rate of rise of insulin resistance is greater. Conversely, diabetes risk should increase for any given rate of rise of insulin resistance if carrying capacity is lower.

Minor Comments

L. 58, "glucose exceeds 7 mM": should be "fasting glucose"

L. 99, "unmedicated": Was there also no life-style intervention (diet and/or exercise)?

L. 59 - 60, "Since type-2 diabetes is associated with morbidity and mortality": Prediabetes is also associated with morbidity and mortality. The main reason for treating prediabetes is that it is much easier to reverse than diabetes.

L. 63, "removing glucose from the blood": add "and suppressing release from the liver".

L. 63, "each unit of insulin": Delete "each unit of".

L. 277, Table 1: Say here or at line 272 of the text that the q_i are the quartiles. The phrase "mean risk factor per ratio" was unclear to me.

L. 300, "Some I conditions": Change to "Some insulin conditions" as stated in the rebuttal.

L. 336: Say "the simplified Topp model" rather than "the Topp model"

Footnote after L. 423: "HOMA-B is proportional to 1/I" should be "HOMA-S is proportional to 1/I".

L. 462: I think Ref #26 should be #29.

L. 484, "Insulin is secreted by beta cells with mass beta cell mass ": should be "Insulin is secreted by beta cells with functional mass ".

L. 592, 597: Which equation, #16 or #17, was used in to make Fig. 4H?

L. 845, legend for Fig. 4, panel F: Say "the simplified Topp model" rather than "the Topp model"

Reviewer #2: None.

Reviewer #3: I appreciate the authors’ efforts in comparing HOMA-C and HOMA-B. However, it is not demonstrated whether the conclusion drawn from HOMA-C in Figure 1 could (or not) be obtained using HOMA-B. While Figure 2 highlights that HOMA-C is a stronger predictor of diabetes transition risk, the comparison of correlations with transition risk alone does not convincingly establish that HOMA-C reflects the concept of “maximal carrying capacity.” In addition, the bootstrapping method used in Figure 2 is not clearly explained and may confuse readers.

Most importantly, the statement “In order to remain roughly constant in an individual, these tests need to vary in a way that keeps HOMA-C constant” suggests that the authors expect HOMA-C to remain relatively stable within individuals, consistent with its proposed definition. However, given the formula for HOMA-C, it is not clear whether this stability can be achieved in practice. I recommend that the authors present individual trajectories of HOMA-C during disease progression. Demonstrating that HOMA-C shows only slight variation within individuals from the prediabetic stage onward would strengthen the argument and provide reassurance to readers regarding its biological interpretation.

**********

**Have the authors made all data and (if applicable) computational code underlying the findings in their manuscript fully available?**

The PLOS Data policy requires authors to make all data and code underlying the findings described in their manuscript fully available without restriction, with rare exception (please refer to the Data Availability Statement in the manuscript PDF file). The data and code should be provided as part of the manuscript or its supporting information, or deposited to a public repository. For example, in addition to summary statistics, the data points behind means, medians and variance measures should be available. If there are restrictions on publicly sharing data or code —e.g. participant privacy or use of data from a third party—those must be specified.

Reviewer #1: Yes

Reviewer #2: Yes

Reviewer #3: Yes

**********

PLOS authors have the option to publish the peer review history of their article (what does this mean?). If published, this will include your full peer review and any attached files.

If you choose “no”, your identity will remain anonymous but your review may still be made public.

**Do you want your identity to be public for this peer review?**  For information about this choice, including consent withdrawal, please see our Privacy Policy.

Reviewer #1: **Yes:** Arthur Sherman

Reviewer #2: No

Reviewer #3: No

[NOTE: If reviewer comments were submitted as an attachment file, they will be attached to this email and accessible via the submission site. Please log into your account, locate the manuscript record, and check for the action link "View Attachments". If this link does not appear, there are no attachment files.]

**Figure resubmission:**

**Reproducibility:**

To enhance the reproducibility of your results, we recommend that authors of applicable studies deposit laboratory protocols in protocols.io, where a protocol can be assigned its own identifier (DOI) such that it can be cited independently in the future. Additionally, PLOS ONE offers an option to publish peer-reviewed clinical study protocols. Read more information on sharing protocols at https://plos.org/protocols?utm_medium=editorial-email&utm_source=authorletters&utm_campaign=protocols

# Author comment: Author response to Decision Letter 2

**DOI:** 10.1371/journal.pcbi.1014589.r004

9 Dec 2025

Attachment Submitted filename: ResponseLetter Homa-C.pdf

# Peer review: Decision Letter 2

**DOI:** 10.1371/journal.pcbi.1014589.r005

**Authors:** Mark Alber, Sunil Laxman

31 Dec 2025

Quantification of beta cell carrying capacity in prediabetes

PLOS Computational Biology

Dear Dr. Woller,

Thank you for submitting your manuscript to PLOS Computational Biology. After careful consideration, we feel that it has merit but does not fully meet PLOS Computational Biology's publication criteria as it currently stands. Therefore, we invite you to submit a revised version of the manuscript that addresses the points raised during the review process.

Please submit your revised manuscript by Mar 02 2026 11:59PM. If you will need more time than this to complete your revisions, please reply to this message or contact the journal office at ploscompbiol@plos.org. When you're ready to submit your revision, log on to https://www.editorialmanager.com/pcompbiol/ and select the 'Submissions Needing Revision' folder to locate your manuscript file.

Please include the following items when submitting your revised manuscript:

* A letter that responds to each point raised by the editor and reviewer(s). You should upload this letter as a separate file labeled 'Response to Reviewers'. This file does not need to include responses to formatting updates and technical items listed in the 'Journal Requirements' section below.

* A marked-up copy of your manuscript that highlights changes made to the original version. You should upload this as a separate file labeled 'Revised Manuscript with Track Changes'.

* An unmarked version of your revised paper without tracked changes. You should upload this as a separate file labeled 'Manuscript'.

If you would like to make changes to your financial disclosure, competing interests statement, or data availability statement, please make these updates within the submission form at the time of resubmission. Guidelines for resubmitting your figure files are available below the reviewer comments at the end of this letter

We look forward to receiving your revised manuscript.

Kind regards,

Sunil Laxman, PhD

Academic Editor

PLOS Computational Biology

Mark Alber

Section Editor

PLOS Computational Biology

**Additional Editor Comments:**

There remain some key points of concern. One is related to the text itself - where some changes (indicated in the response letter) do not manifest in the manuscript text, and also includes one missing reference, and one incorrect statement related to a reference. It is essential for this to be corrected.

The second is related to valid concerns raised by one reviewer, regarding bench marking or comparing against HOMA-B. Given that HOMA-B is widely used for b-cell insulin producing capacity, the reviewer raises valid concerns on the need for some comparison of HOMA-C with HOMA-B, to allow readers to assess whether this index provides a useful, alternate representation of b-cell carrying capacity. It will be critical to provide some feedback on this point - potentially in the results section, but more extensively in the discussion.

**Reviewers' comments:**

Reviewer's Responses to Questions

**Comments to the Authors:**

**Please note here if the review is uploaded as an attachment.**

Reviewer #1: Almost all the requests raised in the second review have now been answered. However, two of the claimed changes do not actually appear in the MS, as listed below, along with one other minor correction.

1. “Models without a carrying-capacity constraint ... (9. 52), as shown in Fig. S5”

These sentences are not in the MS. Similar words are found at l. 425 - 426 but call out Fig. S6, not S5, which seems correct:

“Without the constraint on the timescales of IR rise, prediabetes arises in additional models (9,52) as shown in Fig. S6.”

2. "Added this reference and example in the Discussion (L. 471)".

The reference (Ohn et al, PMID: 26577716) was not added.

3. Hypertrophy vs. hyperplasia. L. 77 - 80: "Current thought is that until adulthood mass change is mainly due to hypertrophy (21)":

Ref. 21 says the opposite, that hyperplasia is dominant until adulthood.

Reviewer #3: The authors use HOMA-C to define “beta-cell carrying capacity,” but report that HOMA-C has higher intra- and inter-CV compared with HOMA-B (see the caption of Figure S4). This raises doubts about whether readers will accept an unstable index as a valid representation of beta-cell carrying capacity.

Furthermore, the authors have not demonstrated whether the conclusions drawn from HOMA-C in Figure 1 could also, or could not, be obtained using HOMA-B. This avoidance reinforces my concern about whether the proposed index HOMA-C meaningfully contributes to the field.

**********

**Have the authors made all data and (if applicable) computational code underlying the findings in their manuscript fully available?**

The PLOS Data policy requires authors to make all data and code underlying the findings described in their manuscript fully available without restriction, with rare exception (please refer to the Data Availability Statement in the manuscript PDF file). The data and code should be provided as part of the manuscript or its supporting information, or deposited to a public repository. For example, in addition to summary statistics, the data points behind means, medians and variance measures should be available. If there are restrictions on publicly sharing data or code —e.g. participant privacy or use of data from a third party—those must be specified.

Reviewer #1: Yes

Reviewer #3: Yes

**********

PLOS authors have the option to publish the peer review history of their article (what does this mean?). If published, this will include your full peer review and any attached files.

If you choose “no”, your identity will remain anonymous but your review may still be made public.

**Do you want your identity to be public for this peer review?**  For information about this choice, including consent withdrawal, please see our Privacy Policy.

Reviewer #1: **Yes:** Arthur Sherman

Reviewer #3: No

[NOTE: If reviewer comments were submitted as an attachment file, they will be attached to this email and accessible via the submission site. Please log into your account, locate the manuscript record, and check for the action link "View Attachments". If this link does not appear, there are no attachment files.]

**Figure resubmission:**

**Reproducibility:**

?>

# Author comment: Author response to Decision Letter 3

**DOI:** 10.1371/journal.pcbi.1014589.r006

8 Apr 2026

Attachment Submitted filename: Response Letter HOMA C March 2026.docx

# Peer review: Decision Letter 3

**DOI:** 10.1371/journal.pcbi.1014589.r007

**Authors:** Mark Alber, Sunil Laxman

29 May 2026

PCOMPBIOL-D-24-00920R3

Quantification of beta-cell carrying capacity in prediabetes

PLOS Computational Biology

Dear Dr. Woller,

Thank you for submitting your extensively revised manuscript to PLOS Computational Biology. After careful consideration, we feel that it has merit but does not fully meet PLOS Computational Biology's publication criteria as it currently stands. Therefore, we invite you to submit a revised version of the manuscript that addresses the points raised during the review process.

Please submit your revised manuscript by Jul 29 2026 11:59PM. If you will need more time than this to complete your revisions, please reply to this message or contact the journal office at ploscompbiol@plos.org. When you're ready to submit your revision, log on to https://www.editorialmanager.com/pcompbiol/ and select the 'Submissions Needing Revision' folder to locate your manuscript file.

Please include the following items when submitting your revised manuscript:

* A letter that responds to each point raised by the editor and reviewer(s). You should upload this letter as a separate file labeled 'Response to Reviewers'. This file does not need to include responses to formatting updates and technical items listed in the 'Journal Requirements' section below.

* A marked-up copy of your manuscript that highlights changes made to the original version. You should upload this as a separate file labeled 'Revised Manuscript with Track Changes'.

* An unmarked version of your revised paper without tracked changes. You should upload this as a separate file labeled 'Manuscript'.

If you would like to make changes to your financial disclosure, competing interests statement, or data availability statement, please make these updates within the submission form at the time of resubmission. Guidelines for resubmitting your figure files are available below the reviewer comments at the end of this letter.

As the corresponding author, your ORCID iD is verified in the submission system and will appear in the published article. PLOS supports the use of ORCID, and we encourage all coauthors to register for an ORCID iD and use it as well. Please encourage your coauthors to verify their ORCID iD within the submission system before final acceptance, as unverified ORCID iDs will not appear in the published article. Only the individual author can complete the verification step; PLOS staff cannot verify ORCID iDs on behalf of authors.

We look forward to receiving your revised manuscript.

Kind regards,

Sunil Laxman, PhD

Academic Editor

PLOS Computational Biology

Mark Alber

Section Editor

PLOS Computational Biology

**Additional Editor Comments:**

Given the bench marking required, this manuscript will mainly just require a presentation of HOMA-C compared to HOMA-B, and a discussion on limitations compared to other methods, and how this method remains an alternate, yet useful approach.

**Reviewers' comments:**

Reviewer's Responses to Questions

**Comments to the Authors:**

**Please note here if the review is uploaded as an attachment.**

Reviewer #1: I am satisfied with the changes, no further comments.

Reviewer #3: While the authors argue that HOMA-C is “stable” based on the ratio of intra- to inter-individual variability, I remain unconvinced that this supports its interpretation as a meaningful physiological construct. The reported coefficient of variation (CV intra ≈ 0.25 vs CV inter ≈ 0.75) does not by itself establish biological stability, as similar behavior can arise from mathematically constrained or model-derived quantities. More importantly, the central concern raised previously remains insufficiently addressed: it is still unclear whether HOMA-C provides information beyond that already contained in HOMA-B. Based on the revised figures (including Fig. S1), the qualitative conclusions drawn from HOMA-C in Figure 1, appear reproducible using HOMA-B alone. If so, HOMA-C may largely represent a reparameterization of the same fasting glucose–insulin relationship rather than a distinct or independently informative metric. Finally, the claim that HOMA-C reflects an intrinsic “carrying capacity” of beta-cell function remains conceptually problematic, as the quantity is derived from state-dependent variables (fasting glucose and insulin) and may therefore reflect the current metabolic environment rather than a fixed physiological limit. I encourage the authors to temper the interpretation of HOMA-C accordingly.

**********

**Have the authors made all data and (if applicable) computational code underlying the findings in their manuscript fully available?**

The PLOS Data policy requires authors to make all data and code underlying the findings described in their manuscript fully available without restriction, with rare exception (please refer to the Data Availability Statement in the manuscript PDF file). The data and code should be provided as part of the manuscript or its supporting information, or deposited to a public repository. For example, in addition to summary statistics, the data points behind means, medians and variance measures should be available. If there are restrictions on publicly sharing data or code —e.g. participant privacy or use of data from a third party—those must be specified.

Reviewer #1: Yes

Reviewer #3: Yes

**********

PLOS authors have the option to publish the peer review history of their article (what does this mean?). If published, this will include your full peer review and any attached files.

If you choose “no”, your identity will remain anonymous but your review may still be made public.

**Do you want your identity to be public for this peer review?**  For information about this choice, including consent withdrawal, please see our Privacy Policy.

Reviewer #1: **Yes:** Arthur Sherman

Reviewer #3: No

[NOTE: If reviewer comments were submitted as an attachment file, they will be attached to this email and accessible via the submission site. Please log into your account, locate the manuscript record, and check for the action link "View Attachments". If this link does not appear, there are no attachment files.]

**Figure resubmission:**

**Reproducibility:**

To enhance the reproducibility of your results, we recommend that authors of applicable studies deposit laboratory protocols in protocols.io, where a protocol can be assigned its own identifier (DOI) such that it can be cited independently in the future. Additionally, PLOS ONE offers an option to publish peer-reviewed clinical study protocols. Read more information on sharing protocols at https://plos.org/protocols?utm_medium=editorial-email&utm_source=authorletters&utm_campaign=protocols

# Author comment: Author response to Decision Letter 4

**DOI:** 10.1371/journal.pcbi.1014589.r008

15 Jul 2026

Attachment Submitted filename: Response Letter HOMA-C 9_07_2026.docx

# Editor report: Decision Letter 4

**DOI:** 10.1371/journal.pcbi.1014589.r009

**Authors:** Mark Alber, Sunil Laxman

17 Jul 2026

Dear Dr Woller,

We are pleased to inform you that your manuscript 'Quantification of beta-cell carrying capacity in prediabetes' has been provisionally accepted for publication in PLOS Computational Biology.

Before your manuscript can be formally accepted you will need to complete some formatting changes, which you will receive in a follow up email. A member of our team will be in touch with a set of requests.

Please note that your manuscript will not be scheduled for publication until you have made the required changes, so a swift response is appreciated.

IMPORTANT: The editorial review process is now complete. PLOS will only permit corrections to spelling, formatting or significant scientific errors from this point onwards. Requests for major changes, or any which affect the scientific understanding of your work, will cause delays to the publication date of your manuscript.

Should you, your institution's press office or the journal office choose to press release your paper, you will automatically be opted out of early publication. We ask that you notify us now if you or your institution is planning to press release the article. All press must be co-ordinated with PLOS.

Thank you again for supporting Open Access publishing; we are looking forward to publishing your work in PLOS Computational Biology.

Best regards,

Sunil Laxman, PhD

Academic Editor

PLOS Computational Biology

Mark Alber

Section Editor

PLOS Computational Biology

***********************************************************

# Editor report: Acceptance letter

**DOI:** 10.1371/journal.pcbi.1014589.r010

**Authors:** Mark Alber, Sunil Laxman

PCOMPBIOL-D-24-00920R4

Quantification of beta-cell carrying capacity in prediabetes

Dear Dr Alon,

I am pleased to inform you that your manuscript has been formally accepted for publication in PLOS Computational Biology. Your manuscript is now with our production department and you will be notified of the publication date in due course.

The corresponding author will soon be receiving a typeset proof for review, to ensure errors have not been introduced during production. Please review the PDF proof of your manuscript carefully, as this is the last chance to correct any errors. Please note that major changes, or those which affect the scientific understanding of the work, will likely cause delays to the publication date of your manuscript.

Soon after your final files are uploaded, unless you have opted out, the early version of your manuscript will be published online. The date of the early version will be your article's publication date. The final article will be published to the same URL, and all versions of the paper will be accessible to readers.

For Research, Software, and Methods articles, you will receive an invoice from PLOS for your publication fee after your manuscript has reached the completed accept phase. If you receive an email requesting payment before acceptance or for any other service, this may be a phishing scheme. Learn how to identify phishing emails and protect your accounts at https://explore.plos.org/phishing.

Thank you again for supporting PLOS Computational Biology and open-access publishing. We are looking forward to publishing your work!

With kind regards,

Sharmila Kamatchi

PLOS Computational Biology | Carlyle House, Carlyle Road, Cambridge CB4 3DN | United Kingdom ploscompbiol@plos.org | Phone +44 (0) 1223-442824 | ploscompbiol.org | @PLOSCompBiol
